# Childhood cancer survival in low- and middle-income countries and the Global South: emerging evidence and critical gaps from a scoping review of observational studies

**DOI:** 10.1016/j.ejcped.2025.100422

**Published:** 2025-12

**Authors:** Marilina Santero, Roberta Ortiz Sequeira, Margarida Cruz Paixao, Maria Muñoz Martinez, Paula Mazorra Roig, Guillermo Chantada, Andres Morales La Madrid, Andre Ilbawi

**Affiliations:** a Universitat Autònoma Barcelona, Barcelona, Spain; b World Health Organization, Department of Non-Communicable Diseases, Disability and Rehabilitation, Geneva, Switzerland; c Pediatric Cancer Center Barcelona, Sant Joan de Déu Children’s Hospital, Barcelona, Spain

**Keywords:** Neoplasms/epidemiology, Pediatric oncology, survival rate, developing countries, health disparities, observational studies as topic

## Abstract

**Background:**

Survival rates for childhood cancer reveal stark global disparities. While over 80 % of children survive in high-income countries (HICs), outcomes remain significantly lower in low- and middle-income countries (LMICs), where the burden is also higher. This study synthesizes observational data on survival outcomes for the six WHO Global Initiative for Childhood Cancer (GICC) index cancers in LMICs, aiming to establish survival estimates, identify key determinants, and assess data limitations.

**Methods:**

Following JBI and PRISMA-ScR guidelines, we conducted a scoping review searching MEDLINE, WHO Global Index Medicus, and EMBASE for observational studies published since 2013. Studies included children aged 0–19 diagnosed with acute lymphoblastic leukemia, Burkitt lymphoma, Hodgkin lymphoma, low-grade glioma, retinoblastoma, or Wilms tumor in LMICs.

**Results:**

From 6358 records, 196 studies were included. Most (72.9 %) were retrospective cohorts; 71.9 % were single-institution studies. The most frequently reported cancers were acute lymphoblastic leukemia (35.2 %) and Wilms tumor (29.1 %). Mean reported overall survival varied widely, from 62.5 % for Burkitt lymphoma (range 20.0–92.0 %) to 78.6 % for Hodgkin lymphoma (range 40.0–96.6 %). Median follow-up was often poorly reported. Socioeconomic barriers, limited healthcare access, and diagnostic delays were common determinants of poor outcomes. Only 10 % of studies referenced hospital-based registries, and fewer than 5 % used population-based data, highlighting critical data gaps.

**Conclusions:**

This review underscores emerging evidence and persistent limitations in childhood cancer survival data from LMICs. The predominance of single-center, retrospective studies indicates a need for more standardized, collaborative research.

## Highlights

• Review of 196 studies on childhood cancer survival in 38 LMICs (2013–2024).
• Assessed survival for six WHO GICC index cancers across diverse settings.
• Found large survival disparities, e.g., 20–92 % for Burkitt, 40–96.6 % for Hodgkin.
• Few studies used population-based data or cancer registries.
• Only 25 % explored socioeconomic factors affecting survival outcomes.

## 1 Introduction

In 2022, more than 275,000 children and adolescents (aged 0–19 years) were diagnosed with cancer worldwide and more than 105,000 lost their lives to the disease [1]; however, this is likely an underestimation due to diagnostic challenges in many countries. Almost 85 % of cases occurred in low- and middle-income countries (LMICs). By 2040, the global burden is expected to nearly double, with the steepest increases projected in LMICs, exacerbating existing health inequities [2].

To address this unequal burden, the WHO launched the Global Initiative for Childhood Cancer (GICC) in 2018, with over 200 partners, including St. Jude Children’s Research Hospital, IARC, SIOP, and CCI 3, 4. The initiative aims to achieve at least 60 % survival worldwide by 2030, focusing on six highly curable, high-priority, index cancers: acute lymphoblastic leukemia (ALL), the most prevalent childhood cancer worldwide; Burkitt lymphoma (BL), common in LMICs; Hodgkin’s lymphoma (HL), frequently affecting adolescents; retinoblastoma, highly curable when diagnosed early; Wilms tumor (WT), a kidney cancer requiring multidisciplinary care; and low-grade glioma (LGG), demanding robust systems for care and treatment. The GICC has been successfully implemented for more than five years using a systematic approach, the CureAll framework [3], and it is now active in more than 80 countries 5, 6, 7.

While survival rates have significantly improved in the last few decades in most high income countries (HICs) – exceeding 80 % five-year survival for many cancer types 8, 9, 10 – many LMICs still report alarmingly low survival rates, ranging from 5 % to 60–70 % 11, 12, 13, 14, primarily due to delayed diagnosis, treatment abandonment, limited access to essential medicines or supportive care, and systemic barriers in health service delivery 15, 16, 17. Despite broad recognition of these disparities, there remains limited and fragmented evidence to support consistent survival estimates in LMICs, as well as insufficient understanding of the contextual factors driving these outcomes.

In this context, this study aims to identify and synthesize the existing evidence from observational studies on childhood cancer survival in LMICs, with the purpose of estimating survival rates, identifying potential determinants, recognizing variations across different settings and understanding data limitations in LMICs. Addressing these gaps will provide valuable insights to measure progress, guide targeted interventions and support evidence-informed policy development.

## 2 Methods

This is a scoping review guided by the Manual for the Synthesis of Evidence of the Joanna Briggs Institute (JBI) [18]. The results are reported using the PRISMA extension for scoping reviews (PRISMA-ScR) checklist [19], as well as the methodology proposed by Global Evidence Mapping Initiative (GEM) [20]. PRISMA checklist is available in Supplemental Table S1. The protocol study was prospectively registered on the OSF Registries on 20 December 2024 [21].

### 2.1 Eligibility criteria

We used the PCC (Population, Concept and Context) framework to guide our review question [18]*.* See Supplemental Table S2 for more details on inclusion and exclusion criteria.

**Population**

Included studies focused on children (0–19 years) diagnosed with one of the six WHO GICC index cancers: ALL, BL, HL, LGG, retinoblastoma, or WT. Studies were excluded if they combined adult data, involved major comorbidities (e.g., HIV, TB, Down syndrome), or focused on refractory/relapsed tumors.

**Concept**

Included studies reported survival outcomes such as overall survival (OS), event-free survival (EFS), or median survival at various time points (1–5 + years).

**Context**

We included data from LMICs published from January 1, 2013, to July 31, 2024. LMIC classification was based on the latest World Bank criteria [22]. Additionally, we included countries from the Global South [23], a term that encompasses nations sharing similar socioeconomic and political characteristics, often associated with lower development levels and historical experiences of colonialism and economic dependency. While not strictly geographical, the Global South generally includes countries in Africa, Asia, Latin America, and Oceania and were analyzed as UMICs (see Supplemental Table S3). While the primary focus of our review is LMICs and the Global South, we included studies from Uruguay and Chile specifically because these countries are commonly considered part of the Global South, despite being classified as high-income according to some economic indicators.

### 2.2 Types of studies

Eligible studies were observational, including cohort (prospective/retrospective), case-control, and cross-sectional studies with survival data. Patient registry and ecological studies were included if relevant. Systematic reviews (SR) were eligible only if based on observational data. Clinical studies reflecting real-world practice were also considered observational in applicable cases.

### 2.3 Data sources and search strategy

We searched the following three databases: MEDLINE (via PubMed), WHO GLOBAL INDEX MEDICUS (GIM), and EMBASE. For detailed search strategies, see Supplemental Table S4. While a grey literature search was not performed, references of included studies were scanned to identify any empirical research satisfying inclusion criteria that may be missed by the search strategy.

### 2.4 Screening

Following the search, all identified records were collated and uploaded into Rayyan [24]. After removing duplicates, at least two reviewers screened independently by title and abstract the search results to identify potentially eligible studies after an initial calibration of 100 references. Where there was disagreement, a third reviewer resolved any conflict. Later, two reviewers independently confirmed eligibility based on the full-text assessment of the potentially relevant articles. Reasons for exclusion in full text assessment that do not meet the inclusion criteria were recorded and reported (Supplemental Table S5). Disagreements on study selection and data extraction were solved by consensus and discussion with other reviewers if needed.

### 2.5 Data extraction

Four reviewers (MS, MM, PM, MP) extracted the following variables from the selected articles: a) Study characteristics, including study ID, author, year of publication, title, DOI, country(ies), setting, multi-institutional status, study aim, study design, methodology, time frame, and inclusion criteria; b) Population demographics, such as the number of patients included in the analysis, age, sex, and follow-up details (methods and median follow-up reported); c) Outcome measures, including survival calculations (Kaplan-Meier figures), OS and EFS at different time points (1, 2, 3, 5, and >5 years), continuous OS and EFS, other survival outcomes, and subgroup analyses; d) Additional study details, such as funding sources, conflicts of interest (COI), ethical approval, collaboration with HICs, clinical setting, study/trial context and details, protocol description and references, and use of hospital- or population-based cancer registries. An adaptation of a risk of bias assessment tool previously published by other authors was done and applied to the selected studies [25]. Also, accordingly we conducted a JBI assessment for each of the study designs included [26].

### 2.6 Variables and data analysis

We categorized the studies based on the income level of the countries involved: UMICs, lower-middle-income countries, LICs, and “multicountry” studies that included those involving more than one country with varying income levels. OS, and EFS served as primary outcomes. Data was summarized both narratively and in tables, categorizing studies based on design, population, concept (outcomes), and context. A descriptive approach highlighting regional differences, data limitations, and trends was taken.

### 2.7 Ethics

Ethical approval and informed consent were not required for this study, as it is a scoping review based solely on the analysis of previously published studies.

## 3 Results

**Fig. 1:**
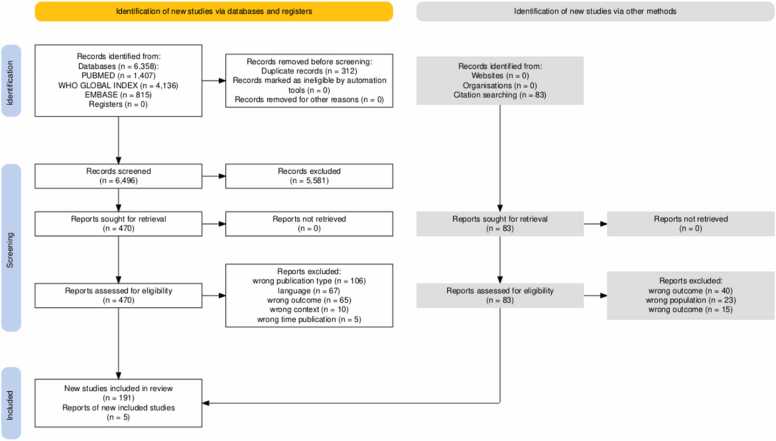
PRISMA flowchart.

Our search yielded 6358 references, of which 470 were assessed for eligibility through full-text review, with 196 studies 14, 17, 25, 27, 28, 29, 30, 31, 32, 33, 34, 35, 36, 37, 38, 39, 40, 41, 42, 43, 44, 45, 46, 47, 48, 49, 50, 51, 52, 53, 54, 55, 56, 57, 58, 59, 60, 61, 62, 63, 64, 65, 66, 67, 68, 69, 70, 71, 72, 73, 74, 75, 76, 77, 78, 79, 80, 81, 82, 83, 84, 85, 86, 87, 88, 89, 90, 91, 92, 93, 94, 95, 96, 97, 98, 99, 100, 101, 102, 103, 104, 105, 106, 107, 108, 109, 110, 111, 112, 113, 114, 115, 116, 117, 118, 119, 120, 121, 122, 123, 124, 125, 126, 127, 128, 129, 130, 131, 132, 133, 134, 135, 136, 137, 138, 139, 140, 141, 142, 143, 144, 145, 146, 147, 148, 149, 150, 151, 152, 153, 154, 155, 156, 157, 158, 159, 160, 161, 162, 163, 164, 165, 166, 167, 168, 169, 170, 171, 172, 173, 174, 175, 176, 177, 178, 179, 180, 181, 182, 183, 184, 185, 186, 187, 188, 189, 190, 191, 192, 193, 194, 195, 196, 197, 198, 199, 200, 201, 202, 203, 204, 205, 206, 207, 208, 209, 210, 211, 212, 213, 214, 215, 216, 217, 218, 219 meeting the inclusion criteria, and being included for data extraction (Fig. 1) [220]. Data extracted spans from 2013 to 2024, with 2023 being the most frequent year (12.1 %).

**Fig. 2:**
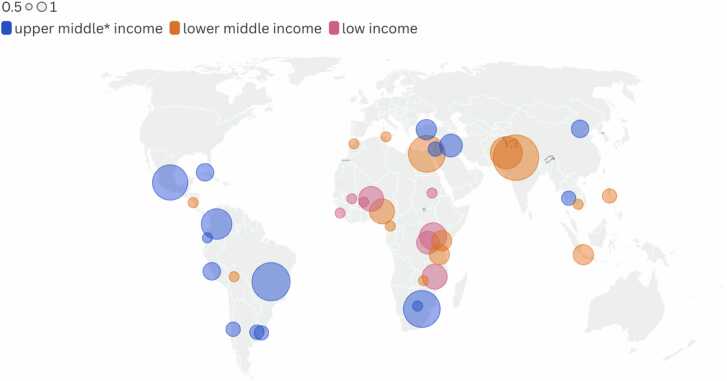
Location of included studies.

The included studies captured data from 38 LMICs (Fig. 2). Eight low-income countries were represented in 23 studies (11.7 %). Fourteen lower-middle-income countries appeared in 67 studies (34.2 %). Sixteen upper-middle-income countries were represented in 82 studies (41.8 %). Additionally, four studies referring to two HICs located in the Global South were included (2 %) 14, 64, 204, 221. Twenty studies (10 %) were conducted in more than one country and categorized as “multicountry”. For details see Supplemental Table S6.

* Includes HICs such as Uruguay and Chile (Global South).

Table 1 describes the main characteristics of the included studies by WHO GICC index cancers. Overall, ALL was the most frequently studied (n = 69, 35.2 %), followed by WT (n = 57, 29.1 %), retinoblastoma (n = 45, 23.0 %), BL (n = 39, 19.9 %), HL (n = 36, 18.4 %), and LGG (n = 32, 16.3 %). Overall, the study population consisted mostly of young children (average age ∼3–8 years) and predominantly male (around 55–73 %).

**Table 1:** Study characteristics (n = 196).

**Cancer type**	**Population**	**Concept**	**Context**
**Acute lymphoblastic leukemia** N = 69 studies 14, 17, 30, 36, 37, 38, 39, 40, 42, 43, 44, 45, 46, 47, 48, 49, 50, 51, 52, 53, 54, 55, 56, 57, 58, 59, 60, 61, 62, 63, 64, 65, 66, 67, 68, 69, 70, 71, 72, 73, 74, 75, 76, 155, 156, 157, 163, 172, 174, 179, 180, 181, 182, 186, 191, 194, 197, 198, 199, 200, 201, 203, 204, 205, 209, 217, 218	Sample size min 20 max 5060 mean 402 Age mean ∼5–7 yrs median ∼5–6 yrs range 1–10 yrs Sex ∼53–73 % male	OS (reported 86 times) mean 64.4 % median 67.6 % range 7.0–95.0 % mean follow 4.3 yrs EFS (reported 58 times) mean 58.2 % median 60.5 % range 18.0–86.2 % mean follow 4.3 yrs	UMIC* (36, 52.2 %) Mexico (7), Brazil (6), Colombia (4), Turkey (3), Cuba (3), Iran (2), Uruguay (2), Chile (1) + others LMIC (24, 34.8 %) India (8), Egypt (7), Pakistan (2), Bolivia (1) + others LIC (5, 7.2 %) Rwanda (3), Botswana (1), Uganda (1) multicountry (4, 5.8 %)
**Burkitt lymphoma and other mature B-cell malignancies** N = 39 studies 14, 17, 25, 32, 33, 34, 35, 131, 133, 154, 155, 156, 157, 164, 178, 179, 186, 188, 191, 192, 195, 198, 199, 200, 202, 204, 207, 217, 218, 239	Sample size min 14 max 609 mean 96 Age mean ∼6.5–8 yrs median ∼7–9 yrs range 1 month–18 yrs Sex ∼60–70 % male	OS (reported 39 times) mean 62.5 % median 63.3 % range 20.0–92.0 % mean follow 3.4 yrs EFS (reported 21 times) mean 61.7 % median 63.3 % range: 27.0–88.0 % mean follow 3.1 yrs	UMIC* (14, 35.9 %) South Africa (4), Brazil (2), Colombia (2), Uruguay (2), Iraq (2), Mexico (1), China (1) LMIC (13, 30.8 %) India (5), Egypt (2), Kenya (2), Nigeria (1), Nicaragua (1), Tanzania (1) LIC (9, 23.1 %) Malawi (4), Uganda (3), Sierra Leone (1), Rwanda (1), Botswana (1) multicountry (3, 7.7 %)
**Hodgkin lymphoma** N = 36 studies 14, 17, 30, 129, 137, 138, 139, 141, 142, 147, 149, 153, 155, 156, 157, 161, 162, 167, 168, 172, 174, 176, 179, 185, 186, 189, 191, 197, 198, 199, 200, 217, 240	Sample size min 5 max 748 mean 179.2 Age mean ∼8 yrs median ∼8.5 yrs range ∼0.25 mo–18 yrs Sex ∼60–65 % male	OS (reported 35 times) mean 78.6 % median 81.5 % range 40.0–96.6 % mean follow 5.7 yrs EFS (reported 19 times) mean 73.7 % median 78.5 % range 46 %–91 %	UMIC* (14, 38.9 %) South Africa (5), Colombia (3), Uruguay (2), Brazil (1), Mexico (1), Turkey (1), Iraq (1) LMIC (14, 38.9 %) Egypt (5), India (4), Pakistan (2), Morocco (1), Tanzania (1), Nicaragua (1) LIC (6, 16.7 %) Rwanda (3), Malawi (1), Botswana (1), Uganda (1) multicountry (2, 5.5 %)
**Low-grade glioma** N = 32 studies 13, 14, 17, 25, 32, 33, 34, 35, 131, 133, 154, 155, 156, 157, 164, 178, 179, 186, 188, 191, 192, 195, 198, 199, 200, 202, 204, 207, 217, 218, 239	Sample size min 3 max 742 mean 170.8 Age mean ∼7.4 yrs median 7.0 yrs range 6 mo–18 yrs Sex ∼55 % male	OS (reported 40 times) mean 66.0 % median 70.6 % range 13.0–93.7 % mean follow 3.8 yrs EFS (reported 4 times) mean 60.0 % median 65.2 % range 41.9–67.9 % mean follow 3.3 yrs	UMIC* (15, 46.9 %) Mexico (3), Colombia (3), Brazil (3), Uruguay (2), Cuba (1), Peru (1), Thailand (1), South Africa (1), LMIC (8, 25.0 %) Egypt (4), Pakistan (1), Nigeria (1), India (1), Venezuela (1), Tunisia (1) LIC (4, 12.5 %) Rwanda (1), Botswana (1), Uganda (1), Sudan (1) Multicountry (5, 15.6 %)
**Retinoblastoma** N = 45 studies 14, 17, 28, 30, 31, 77, 78, 79, 80, 81, 82, 83, 84, 85, 86, 87, 88, 89, 90, 91, 92, 93, 94, 95, 96, 97, 98, 99, 155, 156, 157, 174, 175, 177, 186, 189, 190, 198, 199, 204, 206, 216, 217, 218	Sample size min 3 max 1738 mean 307.2 Age mean ∼3 yrs median ∼2.5 yrs Range 1 mo−12.1 yrs Sex ∼54 % male	OS (reported 61 times) mean 70.0 % median 75.8 % range 0.00 98.0 % mean follow 3.2 yrs EFS (reported 5 times) mean 73.2 % median 71.0 % range 50.0–97.0 % mean follow 4.4 yrs	UMIC* (18, 40.0 %) Brazil (4), Indonesia (3), South Africa (3), Uruguay (2), Argentina (1), China (1), Colombia (1) + others LMIC (12, 26.7 %) Egypt (4), India (3), Pakistan (1), Tanzania (1), Nigeria (1), Cameroon (1), Philippines (1) LIC (7, 15.6 %) Uganda (2), Rwanda (1), Botswana (1), Mali (1), Malawi (1), Burkina Faso (1) multicountry (8, 17.8 %)
**Wilms tumor** N = 57 studies 14, 17, 27, 29, 30, 100, 101, 102, 103, 104, 105, 106, 107, 108, 109, 110, 111, 112, 113, 114, 115, 116, 117, 118, 119, 120, 121, 122, 123, 124, 125, 126, 127, 128, 132, 155, 156, 157, 172, 174, 175, 179, 184, 186, 187, 189, 191, 197, 198, 199, 213, 215, 217, 218, 241	Sample size min 19 max 230 mean 98.7 Age mean ∼8.9 yrs median ∼4.4 yrs range 1 mo−18 yrs Sex ∼60 % male	OS (reported 62 times) mean 68.9 % median 72 % range 12.0–94.6 % mean follow 3.6 yrs EFS (reported 29 times) mean 59.3 % median 66.7 % range 29.0–83.7 % mean follow 3.1 yrs	UMIC* (19, 33.3 %) South Africa (4), Colombia (3), Iran (2), Uruguay (2), Chile (1), Argentina (1), Brazil (1), China (1), Ecuador (1), Jordan (1), Mexico (1), Iraq (1) LMIC (21, 36.8 %) India (5), Egypt (4), Pakistan (4), Nigeria (3), Nicaragua (1), Tanzania (1), Kenya (1), Zambia (1) LIC (9, 15.8 %) Rwanda (3), Malawi (3), Uganda (2), Botswana (1), Tanzania (1) multicountry (8, 14.0 %)

* Includes HICs such as Uruguay and Chile (Global South), EFS: event-free survival, OS: overall survival, LMIC: lower-middle income countries, LIC: low-income countries, max: maximum, min: minimum, mo: months, UMIC: upper-middle income countries, yrs: years.

Across studies, reported OS rates at any different time points (1-, 3-, or 5-year, depending on availability) averaged 62.5 % for BL (range 20.0–92.0 %) and 78.6 % for HL (range 40.0–96.6 %), with EFS rates generally lower (averaged 58.2 % ALL, range 18.0–86.2–73.7 % HL, range 46 %–91 %). Median follow-up time was inadequately reported (less than 40 % of the studies) and ranged between 3 and 5 years when available.

Of the 196 studies reviewed, 164 (83.7 %) were cohort studies, 143 (72.9 %) were retrospective, 18 (9.2 %) were prospective, and 1 (0.5 %) was ambispective in design. Cross-sectional studies accounted for 9 studies (4.6 %), while clinical trials comprised 5 studies (2.5 %). A small number of studies employed alternative designs, including four systematic reviews (2.0 %) and two case-control studies (1.0 %). Additionally, six studies (3.0 %) utilized other designs such as before-after studies, case series, or quasi-experimental approaches. Regarding the type of institutions involved, most studies (n = 141, 71.9 %) were conducted at a single institution, while 47 studies (24.0 %) were multi-institutional.

### 3.1 Outcomes

**Fig. 3:**
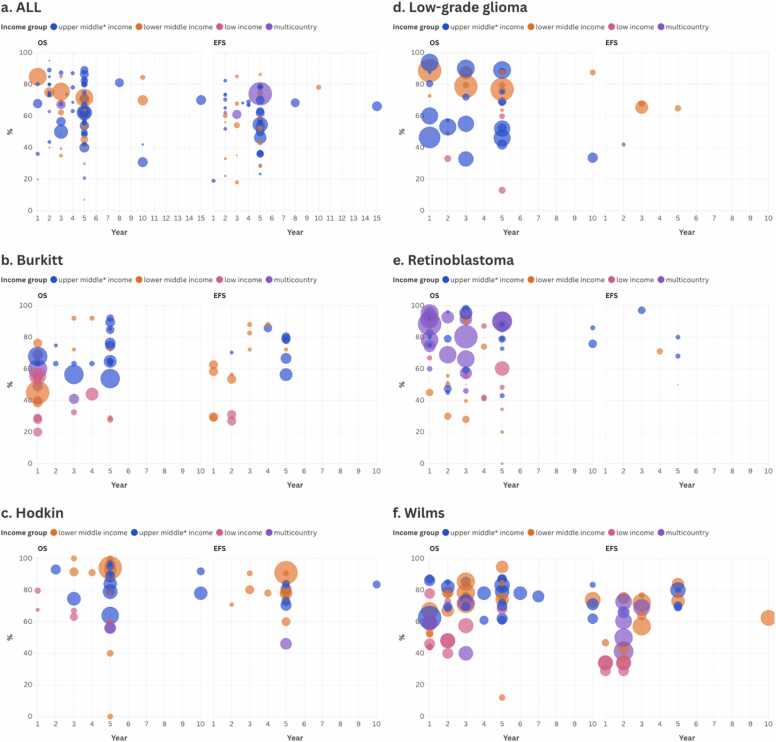
Survival outcomes.

Kaplan-Meier curves were used to visualize survival data in 79.1 % of the studies included. In contrast, only 8.2 % (16 studies) reported OS as a continuous outcome, and just 6.6 % (13 studies) did so for EFS. Fig. 3a–3 f display evidence maps depicting survival outcomes across various time points for each of the six index cancers.

* Includes HICs such as Uruguay and Chile (Global South). ALL: Acute lymphoblastic leukemia

Regarding the availability of OS and EFS data across various time points, the most commonly available form of data is visual-only Kaplan-Meier estimates, especially for 1-year and 2-year OS. Numerical estimates (beyond Kaplan-Meier visuals) are less consistently reported and become increasingly sparse beyond 3 years. For EFS, the data availability is notably lower across all time points especially in longer-term follow-up (≥5 years). Additional insights are provided in Supplemental Table S6, which includes interactive maps.

Generally, UMICs demonstrate higher OS and EFS rates than LICs and lower-middle-income countries. For some cancers, such as HL and WT, survival rates remain relatively high across all income levels. For instance, The visualizations reveal disparities in OS and EFS rates across different cancer types and by World Bank income levels. Generally, UMICs demonstrate higher OS and EFS rates than LICs and lower-middle income countries. For some cancers, such as HL or WT, survival rates remain relatively high across all income levels. For instance, OS rates for HL exceeded 90 % in multiple LMICs, such as Egypt (96.6 %, Sherief 2015), Pakistan (94 %, Mehreen 2019), and India (92.7 %, Trehan 2013), and were also high in UMICs like Morocco (88.3 %, Hessissen 2013) and South Africa (84 %, Geel 2020). However, a persistent gap remains, particularly in regions with limited health system infrastructure. Conversely, survival outcomes for cancers like BL show less consistent patterns across income groups. While some UMICs report relatively high 5-year OS rates — such as Iraq (92 %, Al-Jumaily 2023), Uruguay (89.4 %, Dufort 2021), and South Africa (84.7 %, Kriel 2019) — outcomes in many LMICs and LICs remain significantly lower. For example, OS was 29 % in Tanzania (Saxton 2022), 40 % in Malawi (Westmoreland 2017), and 20 % in Sierra Leone (San Roman 2013). These findings highlight both progress and persistent inequities in outcomes for BL across settings.

Some studies stand out for specific features. For ALL, adapted treatment protocols for resource-limited settings improved outcomes in countries like Mexico, India, Cambodia, Egypt, Turkey, and Peru 40, 43, 47, 53, 61, 182. The largest study, ALL IC-BFM 2002 [50], reported a 74 % 5-year EFS in 5060 patients from 15 countries. Only five studies focused on LIC populations 52, 174, 179, 191, 197. For BL, malnutrition, and infrastructure were key factors; a major multi-institutional trial in sub-Saharan Africa reported a 1-year OS of 60 % [148]. In HL, a large Pakistan study showed 94 % OS and 91 % EFS at 5 years among different chemotherapy regimens [137]; however, these outcomes reflect data from selected tertiary care centers and may not be representative of national survival rates. LGG was often part of broader CNS or pediatric cancer studies; with 44 % reporting outcomes for LGG separately. Brazil´s largest LGG study reported OS rates of 93.7 % (1-year), 90 % (3-year), and 89.0 % (5-year) [164]. Retinoblastoma studies spanned 27 countries and eight international multicountry studies 31, 78, 86, 91, 98, 155, 175, 218 over 4000 patients showed 3-year OS of 57.3 % (LICs), 80.3 % (LMICs), and 91.2 % (UMICs) [91]. For WT, India's largest study reported 3-year OS of 78.3 % (excluding treatment abandonment) and 71.2 % (including it) [112].

### 3.2 Subgroup analyses and potential determinants of survival

Of the 196 studies reviewed, 148 (75.5 %) included subgroup analyses, with only 39 studies (approximately 25 %) specifically examining how socioeconomic determinants of health influenced childhood cancer outcomes. The most reported factors were poverty or low socioeconomic status (18 studies), barriers to healthcare access such as rural residence, displacement, or being part of a vulnerable group (16 studies), and malnutrition or poor nutritional status (12 studies). Other relevant factors included parental education (6 studies), treatment abandonment (9 studies), delays in diagnosis or treatment (7 studies), and health insurance (1 study). Notably, 30 of these 39 studies reported a clear association between one or more of these social determinants and poorer outcomes, such as lower survival, increased treatment abandonment, or early mortality—especially in LMIC.

### 3.3 Conflict of interest, funding and collaborations between countries

Among the 196 studies reviewed, 139 (70.9 %) disclosed conflicts of interest. Of these, 10 (7.2 %) reported specific conflicts such as consultancy roles, employment affiliations and funding from private companies (e.g. pharmaceutical and consultancy companies). In terms of funding, at least 60 studies (30 %) received external support and 20 % did not provide clear funding information. Roughly 28 % of the studies featured clear collaborations with HICs, involving co-authorship, institutional partnerships, funding, training, or twinning initiatives. An additional 24 % had unclear indications of HICs involvement or did not report sufficient information, and 49 % showed no such collaboration.

### 3.4 Cancer registries

Overall, there is a paucity of clearly reported cancer registries among the studies. Only 10 % of the studies described hospital-based cancer registries (HBCR) (n = 20), and population-based cancer registries (PBCR) were explicitly referenced in fewer than 10 studies (5 %). Notable examples included the Rwanda National Cancer Registry [174], the Botswana Pediatric Oncology [191], the central Tunisian cancer registry [212], and the regional Cancer Population Registry of Cali, Colombia [199]. Some studies referenced institutional or disease-specific databases, such as POND (Pediatric Oncology Networked Database) [63], the POLA registry [55] or GALOP (Grupo de America Latina de Oncologia Pediatrica) and COG (Children's Oncology Group), a platform for performing collaborative clinical studies [90].

### 3.5 Risk of bias

**Fig. 4:**
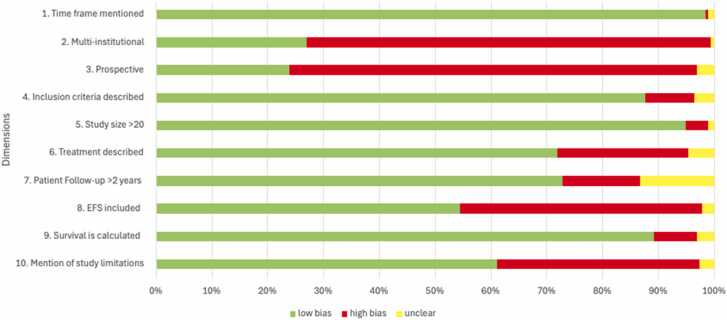
Risk of bias.

A summary of the risk-of-bias assessment is shown in Fig. 4. The main source of bias lay in the fact that the studies were mainly single-center retrospective reviews of relatively small size. Based on our search inclusion criteria, we purposely limited selection bias by excluding studies focusing on specific sub-populations. Limited descriptions of the treatments used, short follow-up time or not reported, and incomplete outcome analyses make reporting bias the principal source of possible biases for the included studies. Detailed results of the bias assessment for the studies included are detailed in Table S7.

## 4 Discussion

### 4.1 Main results

Our scoping review identified nearly 200 studies with observational survival data on the six GICC index cancers in LMICs. Survival rates varied, with mean OS from 62.5 % for BL (range 20.0–92.0 %) to 78.6 % for HL (range 40.0–96.6 %), and mean EFS from 58.2 % for ALL (range 18.0–86.2 %) to 73.7 % for HL (range 46 %–91 %). However, there were wide variations among countries. In addition, the proportion of published studies of each of the tumors according to the country's income level was also different, so aggregated results should be interpreted with caution. Median follow-up was often poorly reported.

A portion of the reviewed studies examined social determinants in childhood cancer, linking factors like poverty, healthcare access, and malnutrition to worse outcomes, particularly in LMICs. An example of socioeconomic barriers from the data is that children from higher socioeconomic groups have better 5-year OS compared to lower groups (91.2 % versus 78.3 % and 78.8 %, p = 0.055). High-risk and undernourished children face higher mortality. Treatment abandonment, often linked to socioeconomic hardship, also significantly increases death risk [42]. These factors show how poverty, poor nutrition, and low socioeconomic status limit access to timely, effective care and worsen outcomes. Another notable finding was the paucity of robust registry data, particularly at the national level. However, some of the few existing national registries report their results in their original language and the results are seldom published in peer-reviewed literature making it challenging to draw conclusions on the existence of cancer registries.

### 4.2 Results in context

More than 1.9 billion children live in LICs and lower-middle-income countries, compared to approximately 450 million children in UMICs [222]. This means that over 80 % of the world’s children are growing up in LICs and lower-middle-income countries —regions where early childhood development, health, and education outcomes are generally worse. While survival indicators may look better in UMICs, these represent a much smaller share of the global child population. As a result, the global picture of child well-being is strongly shaped by conditions in LICs and lower-middle-income countries, where the need for investment and intervention is greatest.

The findings from this scoping review provide essential evidence on childhood cancer outcomes in LMICs, to benchmark progress toward the GICC target, while simultaneously exposing enduring and substantial gaps in the available data. Although most global regions are represented, significant omissions persist. These gaps are unlikely to result from academic oversight alone; rather, they reflect entrenched systemic barriers to reliable health data acquisition, including pervasive underdiagnosis, resource constraints, and inadequate infrastructure 223, 224, 225.

A predominant feature of the current evidence base is its reliance on data derived from tertiary or academic hospitals. Nonetheless, the SIOP Global Mapping Programme, for example, demonstrates that pediatric oncology care in Latin America is markedly centralized: approximately 70 centers manage two-thirds of all pediatric cancer patients, with around 80 % treated in public institutions [226]. This pattern reflects a regional model in which most children are managed in specialized children’s hospitals rather than general cancer centers 227, 228. Although comprehensive data from other continents is limited, it is plausible that similar centralization and dependence on public healthcare systems is present in other LMICs, given shared challenges such as limited resources and the need to concentrate specialized expertise 228, 229. As such, data from tertiary hospitals might represent the reality for a lot of children diagnosed across LMIC and the Global South.

Nevertheless, the absence of robust national cancer registries in many countries continues to impede accurate assessment of outcomes and optimal resource allocation. For example, while Brazil has at least 20 cancer registries, it still lacks a national registry capable of reporting survival estimates for children with cancer [228]. Indeed, based on our results, we consider that exclusive reliance on facility-based registries perpetuates knowledge gaps regarding cancer incidence, access to care, and outcomes among populations not served by major referral centers. High rates of missing data—especially on staging, treatment, and follow-up—further limit the comprehensiveness and utility of HBCRs [230]. For example, recent analyses show that only 25 % of cancer stage fields and as little as 2 % of outcome data fields may be completed, largely due to fragmented data sources and lack of integration with electronic medical records [231].

Addressing these challenges will require harmonization of registry practices, integration with electronic health records, and embedding registries within national health programs to expand coverage and improve data completeness. Investments in training, infrastructure, and long-term sustainability planning are also essential. Strengthening collaboration between ministries of health, academic institutions, and international partners can support the development of robust, standardized systems. Ultimately, while facility-based registries are crucial, their data must be contextualized and complemented by broader, population-based efforts to ensure equitable and accurate representation of the cancer burden. Notably, initiatives such as the IARC-led ChildGICR project are working to address these challenges by strengthening cancer registry capacity and improving the quality and availability of childhood cancer data, particularly in resource-limited countries 232, 233.

### 4.3 Comparative outcomes and inequities

Comparisons of index cancer outcomes between income levels consistently highlight the magnitude of global inequities. For example, survival rates for ALL in HICs often exceed 85 %, whereas reported estimates in our scoping review can be as low as 20–40 % 30, 39, 47, 57, 174, 197, 199 reflecting disparities in access to diagnosis and treatment. On the other hand, for LGG the studies tend to show a narrower range of survival estimates, likely due to the limitations of health system capacities across LMICs.

### 4.4 Determinants of outcomes and research gaps

Most studies stratifying childhood cancer survival in LMICs focus predominantly on classical clinical and epidemiological variables-such as age, sex, and tumor stage, mirroring the approach commonly used in high-income countries. However, these variables do not fully capture the broader determinants that influence outcomes in LMICs, leading to significant research gaps.

Recent systematic reviews and frameworks underscore the multifactorial nature of delays in childhood cancer care in LMICs, identifying determinants such as household income, parental education, travel distance, lack of transportation, reliance on traditional medicine, and health system capacity as critical contributors to delayed diagnosis and treatment initiation [225]. Our review highlights the range and frequency of reported delay-related factors—including socioeconomic barriers, geographic access, and health system limitations. While most included studies did not use standardized delay frameworks, our findings support and complement recent literature by demonstrating how these factors manifest in diverse LMIC settings. This synthesis underscores the urgent need for context-specific strategies to address diagnostic and treatment delays. Socioeconomic context, out-of-pocket health expenditures, and limited availability of pathology and treatment services further exacerbate disparities in outcomes, particularly in rural and underserved populations 234, 235. Consistent with previous research, our findings highlight that having health insurance significantly improves treatment adherence and survival outcomes in pediatric cancer patients by reducing barriers to timely and complete care. A Kenyan study from our review reported that children with health insurance at diagnosis were significantly more likely to complete treatment and achieve event-free survival (53 %), compared to those uninsured (11 %). The HR for treatment failure was 3.1 (95 % CI 1.41–6.60, p = 0.005) for uninsured children versus insured [194]. Similar trends have been described in other LMIC where lack of adequate coverage remains a predictor of poorer outcomes. However, current evidence is still limited, heterogeneous, and largely observational. More robust, multicenter studies are needed to confirm these associations and to guide policies.

Moreover, when discussing the determinants of outcomes, it is crucial to consider co-morbidities, particularly malnutrition and infectious diseases, which are common among children with cancer in LMIC and significantly contribute to poorer treatment outcomes. Additionally, it is important to address myths and miscoceptions about childhood cancer which often results in delays access to biomedical care and increases treatment abandonment, by harnessing the value of people-centred traditional, complementary, and alternative medicine (TCAM) through community awareness and training and by fostering culturally sensitive dialogue, dispelling cancer myths, and promoting integrated care models that respect traditions while ensuring timely, evidence-based treatment is fundamental to address these barriers [references].Despite these challenges, studies evaluating the implementation of context-adapted strategies - such as treatment guidelines tailored to available resources, expanded healthcare coverage, and centralization of care with improvement of care pathways consistently show better patient outcomes [236]. These findings highlight the tangible benefits and feasibility of interventions tailored to local resource constraints and health system realities. Addressing these gaps will require greater emphasis on health systems research, improved documentation of non-clinical determinants, and the development of robust, context-specific data to inform policy and practice. A further observation is the predominance of North American funding and collaboration in studies set in Africa, underscoring the importance of local capacity building and the need for sustainable, regionally led research initiatives [237].

### 4.5 Implications

The findings from the scoping review confirm that significant survival gaps exist across countries based on income strata. The findings provide a robust empirical baseline to track progress, identify barriers and accelerate national response in alignment with the GICC Cure*All* Framework. Based on the observed disparities and service gaps, the study supports several actionable recommendations for policy and practice: strengthening population-based cancer registries to inform planning and resource allocation; expanding access to early diagnosis by strengthening national referral pathways, particularly in rural and underserved areas as well as increasing access to essential treatment services by building primary care providing competencies and establishing share care models to allow treatment adhesion; and addressing socioeconomic barriers—including poverty, travel costs, and health literacy—through multisectoral approaches.

Additionally, the findings reinforce the need to integrate childhood cancer into national cancer control plans and universal health coverage schemes, while investing in the pediatric oncology workforce. The body of evidence will serve as a relevant advocacy tool for the WHO Global Status Report on Cancer 2025, by reinforcing the relevance of including childhood cancer within the broader NCD agenda to accelerate progress toward achieving SDG 3.4 goal of reducing premature mortality by NCDs by one third by 2030. Limitations

By excluding interventional studies, the review provides limited insight into the effectiveness of specific treatments or interventions. Additionally, the heterogeneity in study designs and reporting standards among the included studies may pose challenges for direct comparison and synthesis. A notable limitation is the frequent lack of reporting on median follow-up time in several studies, which may affect the accuracy and comparability of survival estimates. Without consistent follow-up times, it becomes challenging to interpret survival estimates. This limitation may introduce bias, as shorter follow-up periods might underestimate events such as mortality or disease recurrence, while longer follow-ups provide more robust data. While the focus on identifying data gaps is a notable strength, the reliance on published literature may result in the omission of relevant unpublished or region-specific data, potentially underrepresenting certain populations or settings. Finally, when comparing the estimates derived from this review to data from international sources such as IARC or the CONCORD programme [238], it is important to recognize that survival figures based primarily on hospital-based data may be higher than those derived from PBCR, due to selection bias favoring children who are able to access and receive treatment. This highlights the urgent need to expand PBCR to obtain a more accurate and equitable understanding of the global childhood cancer burden.

## 5 **Conclusion**

Our study maps published survival data for the six GICC index cancers across LMICs and the Global South, showcasing significant differences in survival outcomes and variability in data availability and quality. We provide updated survival estimates, highlight key socioeconomic and health system determinants influencing outcomes, and document variations across different income groups and settings. While our findings challenge the assumption that survival data for these cancers are scarce or absent in LMICs, they also underscore the continued need for comprehensive population-based cancer registries, targeted policy interventions, and strengthened health systems. Importantly, these data establish a critical baseline and serve as a progress tracker toward the WHO GICC target of at least 60 % survival globally by 2030. Achieving this goal will require not only improved access to timely diagnosis and treatment but also investment in robust data infrastructure and attention to the social determinants that influence childhood cancer outcomes in LMICs.

## Funding

This research received no specific grant from any funding agency in the public, commercial or not-for-profit sectors.

## CRediT authorship contribution statement

**Maria Muñoz Martinez:** Writing – review & editing, Writing – original draft, Investigation, Formal analysis, Data curation, Conceptualization. **Guillermo Chantada:** Writing – review & editing, Writing – original draft, Validation, Supervision, Methodology, Investigation, Data curation, Conceptualization. **Paula Mazorra Roig:** Writing – review & editing, Writing – original draft, Investigation, Formal analysis, Data curation, Conceptualization. **Andre Ilbawi:** Writing – review & editing, Supervision, Conceptualization. **Andres Morales La Madrid:** Writing – review & editing, Supervision, Conceptualization. **Margarida Cruz Paixao:** Writing – review & editing, Writing – original draft, Validation, Investigation, Formal analysis, Data curation, Conceptualization. **Roberta Ortiz Sequeira:** Writing – review & editing, Writing – original draft, Supervision, Data curation, Conceptualization. **Marilina Santero:** Writing - review & editing, Writing - original draft, Validation, Supervision, Methodology, Investigation, Formal analysis, Data curation, Conceptualization.

## Declaration of Generative AI and AI-assisted technologies in the writing process

During the preparation of this work the authors used ChatGPT and Perplexity in order to improve the language and readability of the manuscript. After using this tool/service, the authors reviewed and edited the content as needed and take full responsibility for the content of the publication.

## Declaration of Competing Interest

The authors declare that they have no known competing financial interests or personal relationships that could have appeared to influence the work reported in this paper.
