# Supplementary material for: Childhood cancer survival in low- and middle-income countries and the Global South: emerging evidence and critical gaps from a scoping review of observational studies
Source: EJC Paediatr Oncol. 2025 Dec;6:None. doi: 10.1016/j.ejcped.2025.100422 (PMC12682699; doi:10.1016/j.ejcped.2025.100422)
Supplement: Table S1 — Supplementary material [file mmc1.docx]

**SUPPORTING INFORMATION MATERIAL**

**Table S1. Preferred Reporting Items for Systematic reviews and Meta-Analyses extension for Scoping Reviews (PRISMA-ScR) Checklist**

| **SECTION** | **ITEM** | **PRISMA-ScR CHECKLIST ITEM** | **REPORTED ON PAGE #** |
| --- | --- | --- | --- |
| **TITLE** | | | |
| Title | 1 | Identify the report as a scoping review. | 1 |
| **ABSTRACT** | | | |
| Structured summary | 2 | Provide a structured summary that includes (as applicable): background, objectives, eligibility criteria, sources of evidence, charting methods, results, and conclusions that relate to the review questions and objectives. | 3 |
| **INTRODUCTION** | | | |
| Rationale | 3 | Describe the rationale for the review in the context of what is already known. Explain why the review questions/objectives lend themselves to a scoping review approach. | 4 |
| Objectives | 4 | Provide an explicit statement of the questions and objectives being addressed with reference to their key elements (e.g., population or participants, concepts, and context) or other relevant key elements used to conceptualize the review questions and/or objectives. | 4 |
| **METHODS** | | | |
| Protocol and registration | 5 | Indicate whether a review protocol exists; state if and where it can be accessed (e.g., a Web address); and if available, provide registration information, including the registration number. | 5 |
| Eligibility criteria | 6 | Specify characteristics of the sources of evidence used as eligibility criteria (e.g., years considered, language, and publication status), and provide a rationale. | 5 |
| Information sources* | 7 | Describe all information sources in the search (e.g., databases with dates of coverage and contact with authors to identify additional sources), as well as the date the most recent search was executed. | 6 |
| Search | 8 | Present the full electronic search strategy for at least 1 database, including any limits used, such that it could be repeated. | 6 |
| Selection of sources of evidence† | 9 | State the process for selecting sources of evidence (i.e., screening and eligibility) included in the scoping review. | 6 |
| Data charting process‡ | 10 | Describe the methods of charting data from the included sources of evidence (e.g., calibrated forms or forms that have been tested by the team before their use, and whether data charting was done independently or in duplicate) and any processes for obtaining and confirming data from investigators. | 7 |
| Data items | 11 | List and define all variables for which data were sought and any assumptions and simplifications made. | 7 |
| Critical appraisal of individual sources of evidence§ | 12 | If done, provide a rationale for conducting a critical appraisal of included sources of evidence; describe the methods used and how this information was used in any data synthesis (if appropriate). | 7 |
| Synthesis of results | 13 | Describe the methods of handling and summarizing the data that were charted. | 7 |
| **RESULTS** | | | |
| Selection of sources of evidence | 14 | Give numbers of sources of evidence screened, assessed for eligibility, and included in the review, with reasons for exclusions at each stage, ideally using a flow diagram. | 7 |
| Characteristics of sources of evidence | 15 | For each source of evidence, present characteristics for which data were charted and provide the citations. | 8 |
| Critical appraisal within sources of evidence | 16 | If done, present data on critical appraisal of included sources of evidence (see item 12). | 11 |
| Results of individual sources of evidence | 17 | For each included source of evidence, present the relevant data that were charted that relate to the review questions and objectives. | 8 |
| Synthesis of results | 18 | Summarize and/or present the charting results as they relate to the review questions and objectives. | 8, 9, 10 |
| **DISCUSSION** | | | |
| Summary of evidence | 19 | Summarize the main results (including an overview of concepts, themes, and types of evidence available), link to the review questions and objectives, and consider the relevance to key groups. | 11 |
| Limitations | 20 | Discuss the limitations of the scoping review process. | 14 |
| Conclusions | 21 | Provide a general interpretation of the results with respect to the review questions and objectives, as well as potential implications and/or next steps. | 15 |
| **FUNDING** | | | |
| Funding | 22 | Describe sources of funding for the included sources of evidence, as well as sources of funding for the scoping review. Describe the role of the funders of the scoping review. | 15 |

JBI = Joanna Briggs Institute; PRISMA-ScR = Preferred Reporting Items for Systematic reviews and Meta-Analyses extension for Scoping Reviews. * Where *sources of evidence* (see second footnote) are compiled from, such as bibliographic databases, social media platforms, and Web sites. † A more inclusive/heterogeneous term used to account for the different types of evidence or data sources (e.g., quantitative and/or qualitative research, expert opinion, and policy documents) that may be eligible in a scoping review as opposed to only studies. This is not to be confused with *information sources* (see first footnote). ‡ The frameworks by Arksey and O’Malley (6) and Levac and colleagues (7) and the JBI guidance (4, 5) refer to the process of data extraction in a scoping review as data charting*.* § The process of systematically examining research evidence to assess its validity, results, and relevance before using it to inform a decision. This term is used for items 12 and 19 instead of "risk of bias" (which is more applicable to systematic reviews of interventions) to include and acknowledge the various sources of evidence that may be used in a scoping review (e.g., quantitative and/or qualitative research, expert opinion, and policy document).

*From:* Tricco AC, Lillie E, Zarin W, O'Brien KK, Colquhoun H, Levac D, et al. PRISMA Extension for Scoping Reviews (PRISMAScR): Checklist and Explanation. Ann Intern Med. 2018;169:467–473. [doi: 10.7326/M18-0850](http://annals.org/aim/fullarticle/2700389/prisma-extension-scoping-reviews-prisma-scr-checklist-explanation).

**Table S2. Eligibility criteria**

|  | Inclusion (all needed) | Exclusion (at least one) |
| --- | --- | --- |
| Population | Population Children aged 0-19 years diagnosed with ALL, Burkitt lymphoma and other mature B cell malignancies, Hodgkin lymphoma, retinoblastoma, Wilms tumor, or low-grade glioma. | Studies focused on other cancers or adult populations, relapsed or recurrence, important comorbidities (e.g HIV, tuberculosis, Down syndrome, etc) |
| Concept | Observational studies reporting survival rates (1, 2, 3, 5, and >5 years post-diagnosis and/or median survival rates. | Studies not reporting survival outcomes or focused on unrelated outcomes. |
| Context | LMICs and Global South; data published between January 1, 2013, and July 31, 2024. | Studies conducted only in HICs or outside the specified timeframe. |
| Study design | Observational studies such as: cohort (prospective/retrospective), case-control, cross-sectional, patient registry studies, ecological studies. SR only if they include observational studies.  Only full text articles. | Clinical trials*, quasiexperimental or other nonobservational designs.  *Clinical studies/experiences from the clinical setting/pragmatic trials/protocols from usual practice (could be considered also observational, should be included) |

**Table S3. Countries generally considered part of the Global South**

| Africa  Angola, Algeria, Benin, Botswana, Burkina Faso, Burundi, Cabo Verde, Cameroon, Chad, Comoros, Congo, Côte d’Ivoire, Egypt, Eritrea, Eswatini, Ethiopia, Gabon, Gambia, Ghana, Guinea, Guinea-Bissau, Equatorial Guinea, Kenya, Lesotho, Liberia, Libya, Madagascar, Malawi, Mali, Mauritania, Mauritius, Morocco, Mozambique, Namibia, Niger, Nigeria, Central African Republic, Democratic Republic of the Congo, Rwanda, São Tomé and Príncipe, Senegal, Seychelles, Sierra Leone, Somalia, South Africa, South Sudan, Sudan, Tanzania, Togo, Tunisia, Uganda, Djibouti, Zambia, Zimbabwe. |
| --- |
| Latin America and the Caribbean  Antigua and Barbuda, Argentina, Bahamas, Barbados, Belize, Bolivia, Brazil, Chile, Colombia, Costa Rica, Cuba, Dominica, Ecuador, El Salvador, Grenada, Guatemala, Guyana, Haiti, Honduras, Jamaica, Mexico, Nicaragua, Panama, Paraguay, Peru, Dominican Republic, Saint Kitts and Nevis, Saint Lucia, Saint Vincent and the Grenadines, Suriname, Trinidad and Tobago, Uruguay, Venezuela. |
| Asia and the Middle East  Afghanistan, Saudi Arabia, Armenia, Azerbaijan, Bangladesh, Bahrain, Myanmar (Burma), Brunei, Bhutan, Cambodia, Qatar, China, North Korea, United Arab Emirates, Philippines, Georgia, India, Indonesia, Iran, Iraq, Jordan, Kazakhstan, Kyrgyzstan, Kuwait, Laos, Lebanon, Malaysia, Maldives, Mongolia, Nepal, Oman, Pakistan, Palestine (West Bank and Gaza), Singapore, Syria, Sri Lanka, Tajikistan, Thailand, Timor-Leste, Turkmenistan, Uzbekistan, Vietnam, Yemen. |
| Oceania  Fiji, Marshall Islands, Solomon Islands, Kiribati, Micronesia, Nauru, Palau, Papua New Guinea, Samoa, Tonga, Tuvalu, Vanuatu. |

**Table S4.** **Search strategy**

| **MEDLINE (1376 references)**  ((cancer OR neoplasms[MeSH] OR neoplasm*) AND (child* OR pediatric OR adolescent OR "0-19 years"))   AND   (survival[MeSH] OR mortality[MeSH] OR survival OR mortality)   AND  (observational OR cohort OR case-control OR cross-sectional OR retrospective OR prospective)   AND  (developing countries[MeSH] OR "low income" OR "middle income" OR LMIC OR "low-resource" OR "Africa" OR "Asia" OR "Latin America" OR "South America")  Search conducted on November 11, 2024 at 10:34 PM  Searcher: Marilina Santero |
| --- |
| **WHO Global Index Medicus (4133 references)**  (Includes studies from regional databases such as LILACS, AIM, IMEMR, WPRIM, and others)  ((cancer OR neoplasm*) AND (child* OR pediatric OR adolescent))   AND   (survival OR mortality OR "survival analysis")  Search conducted on December 20, 2024 at 1:32 PM  Searcher: Marilina Santero |
| **EMBASE (815 references)**  ('cancer'/exp OR 'neoplasm'/exp OR neoplasm* OR cancer) AND ('child'/exp OR 'pediatric'/exp OR 'adolescent'/exp OR '0-19 years' OR child* OR pediatric OR adolescent) AND ('survival'/exp OR 'mortality'/exp OR survival OR mortality) AND ('observational study'/exp OR 'cohort study'/exp OR 'case control study'/exp OR 'cross-sectional study'/exp OR 'retrospective study'/exp OR 'prospective study'/exp OR observational OR cohort OR 'case-control' OR 'cross-sectional' OR retrospective OR prospective) AND ('developing country'/exp OR 'low income'/exp OR 'middle income'/exp OR lmic OR 'low-resource') AND [2013-2025]/py |

**Table S5.** **Excluded studies**

| <https://pubmed.ncbi.nlm.nih.gov/37384861/> |
| --- |
| <https://pubmed.ncbi.nlm.nih.gov/32372372/> |
| <https://pubmed.ncbi.nlm.nih.gov/31352772/> |
| <https://pubmed.ncbi.nlm.nih.gov/34114307/> |
| <https://pubmed.ncbi.nlm.nih.gov/24487917/> |
| <https://pubmed.ncbi.nlm.nih.gov/36445236/> |
| <https://pubmed.ncbi.nlm.nih.gov/29396719/> |
| <https://pubmed.ncbi.nlm.nih.gov/28423236/> |
| <https://pubmed.ncbi.nlm.nih.gov/31172285/> |
| <https://pubmed.ncbi.nlm.nih.gov/38995395/> |
| <https://pubmed.ncbi.nlm.nih.gov/29417460/> |
| <https://pubmed.ncbi.nlm.nih.gov/33974585/> |
| <https://pubmed.ncbi.nlm.nih.gov/26376890/> |
| <https://pubmed.ncbi.nlm.nih.gov/29350457/> |
| <https://pubmed.ncbi.nlm.nih.gov/31184419/> |
| <https://pubmed.ncbi.nlm.nih.gov/19434730/> |
| <https://pubmed.ncbi.nlm.nih.gov/23303533/> |
| <https://pubmed.ncbi.nlm.nih.gov/24347434/> |
| <https://pubmed.ncbi.nlm.nih.gov/24686422/> |
| <https://pubmed.ncbi.nlm.nih.gov/32682835/> |
| <https://pubmed.ncbi.nlm.nih.gov/26612121/> |
| <https://pubmed.ncbi.nlm.nih.gov/25292078/> |
| <https://pubmed.ncbi.nlm.nih.gov/28422613/> |
| <https://pubmed.ncbi.nlm.nih.gov/33357483/> |
| <https://pubmed.ncbi.nlm.nih.gov/27561220/> |
| <https://pubmed.ncbi.nlm.nih.gov/35772043/> |
| <https://pubmed.ncbi.nlm.nih.gov/25871614/> |
| <https://pubmed.ncbi.nlm.nih.gov/29873879/> |
| <https://pubmed.ncbi.nlm.nih.gov/26600901/> |
| <https://pubmed.ncbi.nlm.nih.gov/38363314/> |
| <https://pubmed.ncbi.nlm.nih.gov/36591635/> |
| <https://pubmed.ncbi.nlm.nih.gov/37307212/> |
| <https://pubmed.ncbi.nlm.nih.gov/39281377/> |
| <https://pubmed.ncbi.nlm.nih.gov/30796065/> |
| <https://pubmed.ncbi.nlm.nih.gov/39020265/> |
| <https://pubmed.ncbi.nlm.nih.gov/26274016/> |
| <https://pubmed.ncbi.nlm.nih.gov/37705154/> |
| <https://pubmed.ncbi.nlm.nih.gov/27781380/> |
| <https://pubmed.ncbi.nlm.nih.gov/39688610/> |
| <https://pubmed.ncbi.nlm.nih.gov/39643564/> |
| <https://pubmed.ncbi.nlm.nih.gov/37197142/> |
| <https://pubmed.ncbi.nlm.nih.gov/32823516/> |
| <https://pubmed.ncbi.nlm.nih.gov/33216645/> |
| <https://pubmed.ncbi.nlm.nih.gov/24079452/> |
| <http://imsear.searo.who.int/handle/123456789/79376> |
| <http://dx.doi.org/10.3760/cma.j.cn112140-20230719-00014> |
| <https://pesquisa.bvsalud.org/gim/resource/en/emr-106951> |
| <http://www.scielo.org.ar/scielo.php?script=sci_arttext&pid=S0325-00752014000100009> |
| <http://imsear.searo.who.int/handle/123456789/189718> |
| <http://www.scielo.br/scielo.php?script=sci_arttext&pid=S0004-282X2013000100007> |
| <http://dx.doi.org/10.3760/cma.j.issn.0253-2727.2018.10.010> |
| <https://www.ajol.info/index.php/njp/article/view/127949> |
| <http://www.scielo.cl/scielo.php?script=sci_arttext&pid=S0034-98872019000400437> |
| <http://www1.inca.gov.br/rbc/n_62/v04/pdf/05-artigo-estado-nutricional-e-desfechos-clinicos-em-pacientes-pediatricos-com-leucemia-linfoblastica-aguda.pdf> <https://fi-admin.bvsalud.org/document/view/gtssp> |
| <http://fi-admin.bvsalud.org/document/view/md3x7> |
| <http://dx.doi.org/10.7499/j.issn.1008-8830.2208126> |
| <http://dx.doi.org/10.3760/cma.j.issn.0253-2727.2018.08.007> |
| <http://imsear.searo.who.int/handle/123456789/72006> |
| <https://pesquisa.bvsalud.org/gim/resource/en/wpr-632867> |
| <http://www.scielosp.org/scielo.php?script=sci_arttext&pid=S0036-36342016000200162> |
| <http://dx.doi.org/10.7534/j.issn.1009-2137.2015.01.002> |
| <https://pesquisa.bvsalud.org/gim/resource/en/lil-724708> |
| <http://dx.doi.org/> |
| <http://www.scielo.br/scielo.php?script=sci_arttext&pid=S1807-59322013000200016> |
| <http://dx.doi.org/> |
| <http://www.scielo.br/scielo.php?script=sci_arttext&pid=S1414-462X2013000300007> |
| <https://pesquisa.bvsalud.org/gim/resource/en/emr-206652> |
| <http://www.scielosp.org/scielo.php?script=sci_arttext&pid=S1020-49892024000100806> |
| <http://dx.doi.org/10.19746/j.cnki.issn.1009-2137.2021.05.015> |
| <http://dx.doi.org/10.14791/btrt.2019.7.e36> |
| <http://dx.doi.org/10.7534/j.issn.1009-2137.2016.01.014> |
| <http://dx.doi.org/10.3760/cma.j.issn.0253-2727.2014.04.012> |
| <http://dx.doi.org/10.3760/cma.j.issn.0253-2727.2014.06.005> |
| <http://dx.doi.org/10.3760/cma.j.issn.0253-2727.2014.06.003> |
| <http://dx.doi.org/10.3760/cma.j.issn.0253-2727.2014.04.015> |
| <http://dx.doi.org/10.4143/crt.2022.1618> |
| <http://imsear.searo.who.int/handle/123456789/50453> |
| <http://dx.doi.org/10.19746/j.cnki.issn.1009-2137.2022.02.015> |
| <http://dx.doi.org/10.3760/cma.j.issn.0253-2727.2014.02.011> |
| <http://dx.doi.org/10.19746/j.cnki.issn.1009-2137.2023.03.006> |
| <http://dx.doi.org/10.7534/j.issn.1009-2137.2014.01.017> |
| <http://dx.doi.org/> |
| <http://docs.bvsalud.org/biblioref/coleciona-sus/2017/36453/36453-1648.pdf> |
| <http://dx.doi.org/10.3760/cma.j.issn.0253-2727.2019.08.002> |
| <http://dx.doi.org/10.19746/j.cnki.issn.1009-2137.2022.01.003> |
| <http://dx.doi.org/10.3760/cma.j.cn101070-20191113-01128> |
| <https://pesquisa.bvsalud.org/gim/resource/en/emr-171545> |
| <https://fi-admin.bvsalud.org/document/view/46vcx> |
| <https://revistas.unab.edu.co/index.php/medunab/article/view/2191> <http://fi-admin.bvsalud.org/document/view/j4muf> |
| <https://imsear.searo.who.int/handle/123456789/227986> |
| <https://pesquisa.bvsalud.org/gim/resource/en/biblio-1258776> |
| <https://pesquisa.bvsalud.org/gim/resource/en/emr-141748> |
| <https://rbc.inca.gov.br/index2.php> <https://fi-admin.bvsalud.org/document/view/zngbg> <https://fi-admin.bvsalud.org/document/view/m7nfw> |
| <https://pesquisa.bvsalud.org/gim/resource/en/biblio-1113379> |
| <http://dx.doi.org/10.3760/cma.j.issn.0253-2727.2020.03.003> |
| <http://www.scielo.cl/scielo.php?script=sci_arttext&pid=S0034-98872014000600004> |
| <http://dx.doi.org/10.4103/0366-6999.194661> |
| <http://www.scielosp.org/scielo.php?script=sci_arttext&pid=S1020-49892023000100834> |
| <https://ranc.com.ar/index.php/revista/article/view/156/159> |
| <http://dx.doi.org/10.5045/br.2014.49.1.29> |
| <http://dx.doi.org/10.19746/j.cnki.issn.1009-2137.2023.04.011> |
| <http://dx.doi.org/10.3760/cma.j.cn115356-20220905-00254> |
| <http://www1.inca.gov.br/rbc/n_59/v02/pdf/03b-tendencia-de-mortalidade-por-leucemias-e-linfomas-em-menores-de-20-anos-brasil.pdf> |
| <http://www.teses.usp.br/teses/disponiveis/6/6141/tde-27022019-160813/> |
| <http://dx.doi.org/10.7534/j.issn.1009-2137.2015.05.014> |
| <http://dx.doi.org/> |
| <http://seer.unirio.br/index.php/cuidadofundamental/article/viewFile/7913/pdf_1> <http://seer.unirio.br/index.php/cuidadofundamental/article/view/7913/pdf> |
| <http://www.scielo.br/scielo.php?script=sci_arttext&pid=S1415-790X2024000100400> |
| <http://www.scielo.org.co/scielo.php?script=sci_arttext&pid=S1657-70272017000200006> |
| <http://dx.doi.org/10.3760/cma.j.cn112140-20221005-00853> |
| <http://dx.doi.org/10.19746/j.cnki.issn.1009-2137.2021.03.002> |
| <http://dx.doi.org/10.3760/cma.j.issn.0253-2727.2016.04.002> |
| <http://dx.doi.org/10.3760/cma.j.issn.0253-2727.2013.10.003> |
| <http://dx.doi.org/> |
| <http://dx.doi.org/> |
| <http://dx.doi.org/10.19746/j.cnki.issn.1009-2137.2020.06.019> |
| <http://dx.doi.org/10.3760/cma.j.issn.0253-2727.2013.12.008> |
| <http://dx.doi.org/> |
| <http://dx.doi.org/> |
| <http://dx.doi.org/> |
| <http://dx.doi.org/10.3760/cma.j.cn112138-20190924-00652> |
| <http://dx.doi.org/> |
| <http://dx.doi.org/10.3760/cma.j.issn.1009-9921.2017.07.004> |
| <http://dx.doi.org/10.7534/j.issn.1009-2137.2016.02.018> |
| <http://dx.doi.org/> |
| <http://dx.doi.org/> |
| <http://dx.doi.org/10.19746/j.cnki.issn.1009-2137.2020.04.001> |
| <http://dx.doi.org/> |
| <http://dx.doi.org/10.7499/j.issn.1008-8830.2111064> |
| <http://dx.doi.org/10.3760/cma.j.cn115356-20211012-00238> |
| <http://dx.doi.org/> |
| <http://dx.doi.org/> |
| <http://dx.doi.org/10.19746/j.cnki.issn.1009-2137.2019.04.021> |
| <http://dx.doi.org/10.4143/crt.2023.1205> |
| <http://dx.doi.org/10.7534/j.issn.1009-2137.2018.03.017> |
| <http://dx.doi.org/10.7534/j.issn.1009-2137.2013.03.009> |
| <http://dx.doi.org/> |
| <http://dx.doi.org/10.19746/j.cnki.issn.1009-2137.2023.05.008> |
| <http://dx.doi.org/10.7534/j.issn.1009-2137.2015.03.008> |
| <http://dx.doi.org/10.19746/j.cnki.issn.1009-2137.2021.03.011> |
| <http://dx.doi.org/> |
| <http://dx.doi.org/10.3760/cma.j.issn.1673-4408.2024.01.006> |
| <http://dx.doi.org/> |
| <http://www.scielo.org.mx/scielo.php?script=sci_arttext&pid=S1665-11462014000500002> |
| <http://dx.doi.org/> |
| <http://dx.doi.org/10.3760/cma.j.issn.0253-2727.2022.03.007> |
| <http://dx.doi.org/10.3760/cma.j.issn.0253-2727.2013.12.010> |
| <http://dx.doi.org/10.3760/cma.j.cn101070-20200513-00819> |
| <http://dx.doi.org/10.19746/j.cnki.issn.1009-2137.2019.06.008> |
| <https://www.embase.com/search/results?subaction=viewrecord&id=L2034209938&from=export> U2 - L2034209938 |
| <https://www.embase.com/search/results?subaction=viewrecord&id=L2034210068&from=export> U2 - L2034210068 |
| <https://www.embase.com/search/results?subaction=viewrecord&id=L2036931392&from=export> U2 - L2036931392 |
| <https://www.embase.com/search/results?subaction=viewrecord&id=L645031415&from=export> U2 - L645031415 |
| <https://www.embase.com/search/results?subaction=viewrecord&id=L645031595&from=export> U2 - L645031595 |
| <https://www.embase.com/search/results?subaction=viewrecord&id=L645031647&from=export> U2 - L645031647 |
| <https://www.embase.com/search/results?subaction=viewrecord&id=L2031897457&from=export> U2 - L2031897457 |
| <https://www.embase.com/search/results?subaction=viewrecord&id=L2035786955&from=export> U2 - L2035786955 |
| <https://www.embase.com/search/results?subaction=viewrecord&id=L2028733730&from=export> U2 - L2028733730 |
| <https://www.embase.com/search/results?subaction=viewrecord&id=L643122392&from=export> U2 - L643122392 |
| <https://www.embase.com/search/results?subaction=viewrecord&id=L2018410330&from=export> U2 - L2018410330 |
| <https://www.embase.com/search/results?subaction=viewrecord&id=L2028415004&from=export> U2 - L2028415004 |
| <https://www.embase.com/search/results?subaction=viewrecord&id=L639321698&from=export> U2 - L639321698 |
| <https://www.embase.com/search/results?subaction=viewrecord&id=L639321811&from=export> U2 - L639321811 |
| <https://www.embase.com/search/results?subaction=viewrecord&id=L639322397&from=export> U2 - L639322397 |
| <https://www.embase.com/search/results?subaction=viewrecord&id=L639322651&from=export> U2 - L639322651 |
| <https://www.embase.com/search/results?subaction=viewrecord&id=L639323112&from=export> U2 - L639323112 |
| <https://www.embase.com/search/results?subaction=viewrecord&id=L639323131&from=export> U2 - L639323131 |
| <https://www.embase.com/search/results?subaction=viewrecord&id=L639323913&from=export> U2 - L639323913 |
| <https://www.embase.com/search/results?subaction=viewrecord&id=L639324124&from=export> U2 - L639324124 |
| <https://www.embase.com/search/results?subaction=viewrecord&id=L2019913617&from=export> U2 - L2019913617 |
| <https://www.embase.com/search/results?subaction=viewrecord&id=L2020267885&from=export> U2 - L2020267885 |
| <https://www.embase.com/search/results?subaction=viewrecord&id=L638938891&from=export> U2 - L638938891 |
| <https://www.embase.com/search/results?subaction=viewrecord&id=L2021327985&from=export> U2 - L2021327985 |
| <https://www.embase.com/search/results?subaction=viewrecord&id=L2021328666&from=export> U2 - L2021328666 |
| <https://www.embase.com/search/results?subaction=viewrecord&id=L638055911&from=export> U2 - L638055911 |
| <https://www.embase.com/search/results?subaction=viewrecord&id=L2016085966&from=export> U2 - L2016085966 |
| <https://www.embase.com/search/results?subaction=viewrecord&id=L636404294&from=export> U2 - L636404294 |
| <https://www.embase.com/search/results?subaction=viewrecord&id=L636404501&from=export> U2 - L636404501 |
| <https://www.embase.com/search/results?subaction=viewrecord&id=L636404674&from=export> U2 - L636404674 |
| <https://www.embase.com/search/results?subaction=viewrecord&id=L636404773&from=export> U2 - L636404773 |
| <https://www.embase.com/search/results?subaction=viewrecord&id=L636405201&from=export> U2 - L636405201 |
| <https://www.embase.com/search/results?subaction=viewrecord&id=L636405559&from=export> U2 - L636405559 |
| <https://www.embase.com/search/results?subaction=viewrecord&id=L636405567&from=export> U2 - L636405567 |
| <https://www.embase.com/search/results?subaction=viewrecord&id=L636406618&from=export> U2 - L636406618 |
| <https://www.embase.com/search/results?subaction=viewrecord&id=L636913272&from=export> U2 - L636913272 |
| <https://www.embase.com/search/results?subaction=viewrecord&id=L634123729&from=export> U2 - L634123729 |
| <https://www.embase.com/search/results?subaction=viewrecord&id=L634123791&from=export> U2 - L634123791 |
| <https://www.embase.com/search/results?subaction=viewrecord&id=L634123810&from=export> U2 - L634123810 |
| <https://www.embase.com/search/results?subaction=viewrecord&id=L634124087&from=export> U2 - L634124087 |
| <https://www.embase.com/search/results?subaction=viewrecord&id=L634124149&from=export> U2 - L634124149 |
| <https://www.embase.com/search/results?subaction=viewrecord&id=L634125094&from=export> U2 - L634125094 |
| <https://www.embase.com/search/results?subaction=viewrecord&id=L634130712&from=export> U2 - L634130712 |
| <https://www.embase.com/search/results?subaction=viewrecord&id=L2005737432&from=export> U2 - L2005737432 |
| <https://www.embase.com/search/results?subaction=viewrecord&id=L2013246198&from=export> U2 - L2013246198 |
| <https://www.embase.com/search/results?subaction=viewrecord&id=L2013281031&from=export> U2 - L2013281031 |
| <https://www.embase.com/search/results?subaction=viewrecord&id=L2004021062&from=export> U2 - L2004021062 |
| <https://www.embase.com/search/results?subaction=viewrecord&id=L2002685895&from=export> U2 - L2002685895 |
| <https://www.embase.com/search/results?subaction=viewrecord&id=L627318557&from=export> U2 - L627318557 |
| <https://www.embase.com/search/results?subaction=viewrecord&id=L624178752&from=export> U2 - L624178752 |
| <https://www.embase.com/search/results?subaction=viewrecord&id=L624178870&from=export> U2 - L624178870 |
| <https://www.embase.com/search/results?subaction=viewrecord&id=L624179002&from=export> U2 - L624179002 |
| <https://www.embase.com/search/results?subaction=viewrecord&id=L624179162&from=export> U2 - L624179162 |
| <https://www.embase.com/search/results?subaction=viewrecord&id=L624179215&from=export> U2 - L624179215 |
| <https://www.embase.com/search/results?subaction=viewrecord&id=L624179335&from=export> U2 - L624179335 |
| <https://www.embase.com/search/results?subaction=viewrecord&id=L624179467&from=export> U2 - L624179467 |
| <https://www.embase.com/search/results?subaction=viewrecord&id=L624180008&from=export> U2 - L624180008 |
| <https://www.embase.com/search/results?subaction=viewrecord&id=L624180078&from=export> U2 - L624180078 |
| <https://www.embase.com/search/results?subaction=viewrecord&id=L626457237&from=export> U2 - L626457237 |
| <https://www.embase.com/search/results?subaction=viewrecord&id=L626588839&from=export> U2 - L626588839 |
| <https://www.embase.com/search/results?subaction=viewrecord&id=L643842464&from=export> U2 - L643842464 |
| <https://www.embase.com/search/results?subaction=viewrecord&id=L643842866&from=export> U2 - L643842866 |
| <https://www.embase.com/search/results?subaction=viewrecord&id=L621729379&from=export> U2 - L621729379 |
| <https://www.embase.com/search/results?subaction=viewrecord&id=L616983386&from=export> U2 - L616983386 |
| <https://www.embase.com/search/results?subaction=viewrecord&id=L2001337381&from=export> U2 - L2001337381 |
| <https://www.embase.com/search/results?subaction=viewrecord&id=L2001337401&from=export> U2 - L2001337401 |
| <https://www.embase.com/search/results?subaction=viewrecord&id=L622327711&from=export> U2 - L622327711 |
| <https://www.embase.com/search/results?subaction=viewrecord&id=L620333948&from=export> U2 - L620333948 |
| <https://www.embase.com/search/results?subaction=viewrecord&id=L620335953&from=export> U2 - L620335953 |
| <https://www.embase.com/search/results?subaction=viewrecord&id=L2010115676&from=export> U2 - L2010115676 |
| <https://www.embase.com/search/results?subaction=viewrecord&id=L617538039&from=export> U2 - L617538039 |
| <https://www.embase.com/search/results?subaction=viewrecord&id=L643582350&from=export> U2 - L643582350 |
| <https://www.embase.com/search/results?subaction=viewrecord&id=L614224913&from=export> U2 - L614224913 |
| <https://www.embase.com/search/results?subaction=viewrecord&id=L614246767&from=export> U2 - L614246767 |
| <https://www.embase.com/search/results?subaction=viewrecord&id=L612592385&from=export> U2 - L612592385 |
| <https://www.embase.com/search/results?subaction=viewrecord&id=L612592502&from=export> U2 - L612592502 |
| <https://www.embase.com/search/results?subaction=viewrecord&id=L612592659&from=export> U2 - L612592659 |
| <https://www.embase.com/search/results?subaction=viewrecord&id=L612592690&from=export> U2 - L612592690 |
| <https://www.embase.com/search/results?subaction=viewrecord&id=L612593305&from=export> U2 - L612593305 |
| <https://www.embase.com/search/results?subaction=viewrecord&id=L612593393&from=export> U2 - L612593393 |
| <https://www.embase.com/search/results?subaction=viewrecord&id=L615421172&from=export> U2 - L615421172 |
| <https://www.embase.com/search/results?subaction=viewrecord&id=L627916713&from=export> U2 - L627916713 |
| <https://www.embase.com/search/results?subaction=viewrecord&id=L72175572&from=export> U2 - L72175572 |
| <https://www.embase.com/search/results?subaction=viewrecord&id=L613189643&from=export> U2 - L613189643 |
| <https://www.embase.com/search/results?subaction=viewrecord&id=L613190019&from=export> U2 - L613190019 |
| <https://www.embase.com/search/results?subaction=viewrecord&id=L613190364&from=export> U2 - L613190364 |
| <https://www.embase.com/search/results?subaction=viewrecord&id=L72231458&from=export> U2 - L72231458 |
| <https://www.embase.com/search/results?subaction=viewrecord&id=L72231476&from=export> U2 - L72231476 |
| <https://www.embase.com/search/results?subaction=viewrecord&id=L71962315&from=export> U2 - L71962315 |
| <https://www.embase.com/search/results?subaction=viewrecord&id=L600600334&from=export> U2 - L600600334 |
| <https://www.embase.com/search/results?subaction=viewrecord&id=L71655638&from=export> U2 - L71655638 |
| <https://www.embase.com/search/results?subaction=viewrecord&id=L71655943&from=export> U2 - L71655943 |
| <https://www.embase.com/search/results?subaction=viewrecord&id=L71656091&from=export> U2 - L71656091 |
| <https://www.embase.com/search/results?subaction=viewrecord&id=L71656322&from=export> U2 - L71656322 |
| <https://www.embase.com/search/results?subaction=viewrecord&id=L71656575&from=export> U2 - L71656575 |
| <https://www.embase.com/search/results?subaction=viewrecord&id=L71656606&from=export> U2 - L71656606 |
| <https://www.embase.com/search/results?subaction=viewrecord&id=L71740688&from=export> U2 - L71740688 |
| <https://www.embase.com/search/results?subaction=viewrecord&id=L71505918&from=export> U2 - L71505918 |
| <https://www.embase.com/search/results?subaction=viewrecord&id=L71566245&from=export> U2 - L71566245 |
| <https://www.embase.com/search/results?subaction=viewrecord&id=L71223196&from=export> U2 - L71223196 |
| <https://www.embase.com/search/results?subaction=viewrecord&id=L71223199&from=export> U2 - L71223199 |
| <https://www.embase.com/search/results?subaction=viewrecord&id=L71216000&from=export> U2 - L71216000 |
| <https://www.embase.com/search/results?subaction=viewrecord&id=L71216254&from=export> U2 - L71216254 |
| <https://www.embase.com/search/results?subaction=viewrecord&id=L71216578&from=export> U2 - L71216578 |
| <https://www.embase.com/search/results?subaction=viewrecord&id=L71216772&from=export> U2 - L71216772 |
| <https://www.embase.com/search/results?subaction=viewrecord&id=L71218031&from=export> U2 - L71218031 |
| <https://www.embase.com/search/results?subaction=viewrecord&id=L71047932&from=export> U2 - L71047932 |
| <https://www.embase.com/search/results?subaction=viewrecord&id=L71696165&from=export> U2 - L71696165 |

**S6. Included studies**

1. Abdelmabood S, Fouda AE, Boujettif F, Mansour A. Treatment outcomes of children with acute lymphoblastic leukemia in a middle-income developing country: high mortalities, early relapses, and poor survival. J Pediatr (Rio J). 2020;96: 108–116.
2. Ahmad I, Ghafoor T, Ullah A, Naz S, Tahir M, Ahmed S, et al. Pediatric Acute Lymphoblastic Leukemia: Clinical Characteristics, Treatment Outcomes, and Prognostic Factors: 10 Years’ Experience From a Low- and Middle-Income Country. JCO Glob Oncol. 2023;9: e2200288.
3. Al-Hadad SA, Al-Jadiry MF, Ghali HH, Al-Badri SAF, Al-Saeed RM, Al-Darraji AF, et al. Treatment of childhood acute lymphoblastic leukemia in Iraq: a 17-year experience from a single center. Leuk Lymphoma. 2021;62: 3430–3439.
4. Al-Jumaily U, Habeeb Rjeib HD, Alqanbar MF, Faraj S, Al-Khateeb DA. Improved outcomes of children with Wilms’ tumor in a low-middle-income nation: The contribution of a pediatric oncologist to successful management. Asian J Urol. 2025;12: 93–99.
5. Alakaloko FM, Akinsete AM, Seyi-Olajide JO, Joseph AO, Elebute OO, Ladipo-Ajayi OA, et al. A 5-year multidisciplinary care outcomes in children with wilms’ tumour managed at a tertiary centre: A retrospective observational study. Afr J Paediatr Surg. 2022;19: 83–88.
6. Alert Silva J, Chon Rivas I, Ropero Toirac R. Empleo de la radioterapia en el tratamiento a los tumores del sistema nervioso central en niños y adolescentes. Rev cuba pediatr. 2016; 120–129.
7. Alkayed K, Al Hmood A, Madanat F. Prognostic effect of blood transfusion in children with acute lymphoblastic leukemia. Blood Res. 2013;48: 133–138.
8. Amir A-H, Soheil Z, Mehran K, Esmaeil K, Abolfazl M. Survival rate of childhood leukemia in shiraz, southern iran. Iran J Pediatr. 2013;23: 53–58.
9. Antonisamy N, Boddu D, John R, Korrapolu RSA, Balasubramanian P, Arunachalam AK, et al. The Outcome of Pediatric Philadelphia Chromosome-Positive Acute Lymphoblastic Leukemia: Experience from a Referral Center in South India. Indian J Hematol Blood Transfus. 2024;40: 61–67.
10. Anwar S, Faizan M, Khan S, Tallat N, Saleem M, Khalid A, et al. Five Year Experience of Wilms Tumor at a tertiary care centre, where we stand, a developing country perspective. 2017;11: 1263–1266.
11. Anyanwu L-JC, Atanda AT, Atanda JO. Wilms’ tumour in African children: Can an institutional approach improve outcome? Afr J Paediatr Surg. 2015;12: 7–11.
12. Appeadu-Mensah W, Mdoka C, Alemu S, Yifieyeh A, Kaplamula T, Oyania F, et al. Surgical aspects and outcomes after nephrectomy for Wilms tumour in sub-Saharan Africa: A report from Wilms Africa Phase II-CANCaRe Africa. Pediatr Blood Cancer. 2025;72: e31134.
13. Arazi M, Kfir J, Ahmad A, Foster A, Baum A, Stacey AW, et al. Primary Enucleation for Intraocular Unilateral Retinoblastoma Can Save Life in Lower-Income Settings. Semin Ophthalmol. 2024; 1–5.
14. Aristizabal P, Rivera-Gomez R, Chang A, Ornelas M, Ramirez M, Tamayo G, et al. Childhood Leukemia Survival in the US-Mexico Border: Building Sustainable Leukemia Care Using Health Systems Strengthening Models. JCO Glob Oncol. 2023;9: e2300123.
15. Aronson DC, Hadley GP. Age is not a prognostic factor in children with Wilms tumor beyond stage I in Africa. Pediatr Blood Cancer. 2014;61: 987–989.
16. Arora RS, Kumari R, Adhana A, Tripathi R, Sachdev M, Jain P. Overall and Event Free Survival of Childhood Cancer - Report From a Hospital-based Cancer Registry in Northern India, 2013-21. Indian Pediatr. 2023;60: 531–536.
17. Asfour HY, Khalil SA, Zakaria A-S, Ashraf E-S, Zekri W. Localized Wilms’ tumor in low-middle-income countries (LMIC): how can we get better? J Egypt Natl Canc Inst. 2020;32: 32.
18. Assumpção JG, Paula FDF, Xavier SG, Murao M, de Aguirre JC Neto, Dutra AP, et al. Gene rearrangement study for minimal residual disease monitoring in children with acute lymphocytic leukemia. Rev Bras Hematol Hemoter. 2013;35: 337–342.
19. Atima MO, Idakwo U, Komolafe O, Eisuke S, Shintaro N, Balogun EO, et al. Presentation pattern and survival rate of retinoblastoma following chemotherapy: a prospective study. BMC Pediatr. 2023;23: 538.
20. Bahoush G, Saeedi E. Outcome of Children with Wilms’ Tumor in Developing Countries. J Med Life. 2020;13: 484–489.
21. Barragán-Pérez EJ, Altamirano-Vergara CE, Alvarez-Amado DE, García-Beristain JC, Chico-Ponce-de-León F, González-Carranza V, et al. The Role of Time as a Prognostic Factor in Pediatric Brain Tumors: a Multivariate Survival Analysis. Pathol Oncol Res. 2020;26: 2693–2701.
22. Basbous M, Al-Jadiry M, Belgaumi A, Sultan I, Al-Haddad A, Jeha S, et al. Childhood cancer care in the Middle East, North Africa, and West/Central Asia: A snapshot across five countries from the POEM network. Cancer Epidemiol. 2021;71: 101727.
23. Berry JL, Pike S, Rajagopalan A, Reid MW, Fabian ID, Global Retinoblastoma Study Group. Retinoblastoma Outcomes in the Americas: A Prospective Analysis of 491 Children With Retinoblastoma From 23 American Countries. Am J Ophthalmol. 2024;260: 91–101.
24. Blanco R, Milton W. Aspectos clínicos y sobrevida de los pacientes con retinoblastoma atendidos en el Instituto Nacional de Enfermedades Neoplásicas: Enero 2001- agosto 2007. Acta méd Peru. 2013;30: 69–73.
25. Bordbar M, Jam N, Karimi M, Shahriari M, Zareifar S, Zekavat OR, et al. The survival of childhood leukemia: An 8-year single-center experience. Cancer Rep (Hoboken). 2023;6: e1784.
26. Bouda GC, Traoré F, Couitchere L, Raquin M-A, Guedenon KM, Pondy A, et al. Advanced Burkitt Lymphoma in Sub-Saharan Africa Pediatric Units: Results of the Third Prospective Multicenter Study of the Groupe Franco-Africain d’Oncologie Pédiatrique. J Glob Oncol. 2019;5: 1–9.
27. Brandalise SR, Viana MB, Pinheiro VRP, Mendonça N, Lopes LF, Pereira WV, et al. Shorter Maintenance Therapy in Childhood Acute Lymphoblastic Leukemia: The Experience of the Prospective, Randomized Brazilian GBTLI ALL-93 Protocol. Front Pediatr. 2016;4: 110.
28. Bravo LE, García LS, Collazos P, Aristizabal P, Ramirez O. Descriptive epidemiology of childhood cancer in Cali: Colombia 1977-2011. Colomb Med (Cali). 2013;44: 155–164.
29. Buckle G, Maranda L, Skiles J, Ong’echa JM, Foley J, Epstein M, et al. Factors influencing survival among Kenyan children diagnosed with endemic Burkitt lymphoma between 2003 and 2011: A historical cohort study. Int J Cancer. 2016;139: 1231–1240.
30. Businge L, Hagenimana M, Motlhale M, Bardot A, Liu B, Anastos K, et al. Stage at diagnosis and survival by stage for the leading childhood cancers in Rwanda. Pediatr Blood Cancer. 2024;71: e31020.
31. Causal factors influencing quality of treatment and survival in Wilms Tumor: A retrospective investigation. Pediatric Hematology Oncology Journal. 2023;8: 228–232.
32. Chagaluka G, Paintsil V, Renner L, Weijers J, Chitsike I, Borgstein E, et al. Improvement of overall survival in the Collaborative Wilms Tumour Africa Project. Pediatr Blood Cancer. 2020;67: e28383.
33. Chapman H, Ntemi PS, Gisiri M, Vasudevan L, Kashaigili HJ, Schroeder K. Retrospective analysis of pediatric patients with Burkitt lymphoma treated in Tanzania following the implementation of the 2016 National Treatment Guidelines: Poor outcomes to current standard-of-care therapy. Pediatr Blood Cancer. 2024;71: e31145.
34. Chauhan P, Gupta A, Mandelia A, Yadav S, Rahman K, Nityanand S. Clinical profile, outcome and challenges in the management of pediatric Burkitt lymphoma: a single center experience. Pediatr Hematol Oncol. 2021;38: 305–318.
35. Chen Y, Sun X-F, Zhen Z-J, Wang J, Zhu J, Lu S-Y, et al. Germinal-center type B-cell classification and clinical characteristics of Chinese pediatric diffuse large B-cell lymphoma: a report of 76 cases. Chin J Cancer. 2013;32: 561–566.
36. Cruz PCG, Curtidor LEF, Villabona HMJ, Riveros PM, De Los Reyes Valencia IDC. Estudio de supervivencia de pacientes menores de 18 años de edad con diagnóstico de cáncer atendidos en el Hospital Universitario San Ignacio-Centro Javeriano de Oncología entre octubre de 2010 y marzo de 2016. Univ Med. 2017;58. doi:10.11144/Javeriana.umed58-4.espm
37. Cueva-Arica D. Resultados de dos protocolos de tratamiento para la leucemia linfoide aguda infantil en un hospital peruano. Revista Hematología. 2024;28. doi:10.48057/hematologa.v28i1.568
38. Davidson A, Hendricks M. Experience with B-cell lymphoma at a South African centre in the HIV Era. Transfus Apher Sci. 2013;49: 31–39.
39. DeBoer RJ, Shyirambere C, Driscoll CD, Butera Y, Paciorek A, Ruhangaza D, et al. Treatment of Hodgkin Lymphoma With ABVD Chemotherapy in Rural Rwanda: A Model for Cancer Care Delivery Implementation. JCO Glob Oncol. 2020;6: 1093–1102.
40. Denburg AE, Laher N, Mutyaba I, McGoldrick S, Kambugu J, Sessle E, et al. The cost effectiveness of treating Burkitt lymphoma in Uganda. Cancer. 2019;125: 1918–1928.
41. Duffy C, Graetz DE, Lopez AMZ, Carrillo AK, Job G, Chen Y, et al. Retrospective analysis of outcomes for pediatric acute lymphoblastic leukemia in South American centers. Front Oncol. 2023;13: 1254233.
42. Dufort y Álvarez G. Epidemiología del cáncer en niños y adolescentes en Uruguay: 2008-2012. Un estudio de registro poblacional. Archivos de Pediatría del Uruguay. 2021. doi:10.31134/ap.92.1.3
43. Dujua ACC, Hernandez FG. Survival Outcome of Filipino Children With Acute Lymphoblastic Leukemia Treated With Modified Berlin-Frankfurt-Muenster/Hong Kong Acute Lymphoblastic Leukemia (BFM95/HKALL97) Protocol in a Tertiary General Hospital From January 2005 to December 2009: A Retrospective Cohort Study. J Pediatr Hematol Oncol. 2017;39: e116–e123.
44. Durosinmi MA. Burkitt Lymphoma: A Potentially Curable Childhood Tumour: Experience in Ile-Ife, Nigeria (1986-2014). Ann Health Res. 2016;2: 1–9.
45. Ekuk E, Odongo CN, Tibaijuka L, Oyania F, Egesa WI, Bongomin F, et al. One year overall survival of wilms tumor cases and its predictors, among children diagnosed at a teaching hospital in South Western Uganda: a retrospective cohort study. BMC Cancer. 2023;23: 196.
46. El-Mallawany NK, Wasswa P, Mtete I, Mutai M, Stanley CC, Mtunda M, et al. Identifying opportunities to bridge disparity gaps in curing childhood cancer in Malawi: Malignancies with excellent curative potential account for the majority of diagnoses. Pediatr Hematol Oncol. 2017;34: 261–274.
47. Elhassan MMA, Mohamedani AA, Osman HHM, Yousif NO, Elhaj NM, Qaddoumi I. Patterns, treatments, and outcomes of pediatric central nervous system tumors in Sudan: a single institution experience. Childs Nerv Syst. 2019;35: 437–444.
48. Elzomor H, Taha H, Nour R, Aleieldin A, Zaghloul MS, Qaddoumi I, et al. A multidisciplinary approach to improving the care and outcomes of patients with retinoblastoma at a pediatric cancer hospital in Egypt. Ophthalmic Genet. 2017;38: 345–351.
49. Farrag A, Ghazaly MH, Mohammed K, Volland R, Hero B, Berthold F. Comparing presentations and outcomes of children with cancer: a study between a lower-middle-income country and a high-income country. BMC Pediatrics. 2023;23: 1–12.
50. Fawzy MA, El-Hemaly AI, Awad M, El-Beltagy M, Zaghloul MS, Taha H, et al. Multidisciplinary treatment of pediatric low-grade glioma: Experience of children cancer hospital of Egypt; 2007-2012. Indian J Med Paediatr Oncol. 2018;39: 488–492.
51. Fufa D, Mdoka C, Ayalew M, Khofi H, Amankwah E, Chokwenda N, et al. Effectiveness of a Wilms tumour treatment guideline adapted to local circumstances in sub-Saharan Africa: A report from Wilms Africa Phase II-CANCaRe Africa. Pediatr Blood Cancer. 2024;71: e31300.
52. Gaytan-Morales F, Alejo-Gonzalez F, Reyes-Lopez A, Palomo M, Rodriguez-Romo L, Villareal-Martínez L, et al. Pediatric mature B-cell NHL, early referral and supportive care problems in a developing country. Hematology. 2019;24: 79–83.
53. Geel J, van Zyl A, Plessis J du, Hendricks M, Goga Y, Carr A, et al. Improved survival of children and adolescents with classical Hodgkin lymphoma treated on a harmonised protocol in South Africa. Pediatr Blood Cancer. 2024;71: e30712.
54. Geel JA, Chirwa TC, Rowe B, Eyal KC, Omar F, Stones DK, et al. Treatment outcomes of children with Hodgkin lymphoma between 2000 and 2010: First report by the South African Children’s Cancer Study Group. Pediatr Blood Cancer. 2017;64. doi:10.1002/pbc.26536
55. Geel JA, Eyal KC, Hendricks MG, Myezo KH, Stones DK, Omar F, et al. Prognostic factors affecting survival in children and adolescents with HIV and Hodgkin lymphoma in South Africa. Leukemia & Lymphoma. 2020; 2854–2863.
56. Ghafoor T, Bashir F, Ahmed S, Khalil S, Farah T. Predictors of treatment outcome of Wilms Tumour in low-income country; single centre experience from Pakistan. J Pediatr Urol. 2020;16: 375.e1–375.e7.
57. Ghafoor T. Prognostic factors in pediatric Hodgkin lymphoma: experience from a developing country. Leuk Lymphoma. 2020;61: 344–350.
58. Gibson TN, Beeput S, Gaspard J, George C, Gibson D, Jackson N, et al. Baseline characteristics and outcomes of children with cancer in the English-speaking Caribbean: A multinational retrospective cohort. Pediatric Blood & Cancer. 2018;65: e27298.
59. Girardi F, Allemani C, Coleman MP. Global Trends in Survival From Astrocytic Tumors in Adolescents and Young Adults: A Systematic Review. JNCI Cancer Spectr. 2020;4: kaa049.
60. Girardi F, Allemani C, Coleman MP. Worldwide Trends in Survival From Common Childhood Brain Tumors: A Systematic Review. J Glob Oncol. 2019;5: 1–25.
61. Global Retinoblastoma Study Group. The Global Retinoblastoma Outcome Study: a prospective, cluster-based analysis of 4064 patients from 149 countries. Lancet Glob Health. 2022;10: e1128–e1140.
62. González-Otero A, Menéndez-Veitía A, Machín-García S, Svarch E, Campo-Díaz M, Fernández-Nodarse R, et al. Resultados del tratamiento de la Leucemia Linfoide Aguda del niño en Cuba. Rev Cubana Hematol Inmunol Hemoter. 2014;30: 36–46.
63. Guerrero E, Alvarado R, Urdiales A, Orbe MJ, Navarrete O, Manterola C. Tumor de Wilms: Estudio de Centro Único de Los Andes Ecuatorianos. Serie de Casos con Seguimiento. Int J Morphol. 2020;38: 208–214.
64. Güneş AM, Oren H, Baytan B, Bengoa SY, Evim MS, Gözmen S, et al. The long-term results of childhood acute lymphoblastic leukemia at two centers from Turkey: 15 years of experience with the ALL-BFM 95 protocol. Ann Hematol. 2014;93: 1677–1684.
65. Gupta A, Kapoor G, Jain S, Bajpai R. Absolute Lymphocyte Count Recovery Independently Predicts Outcome in Childhood Acute Lymphoblastic Leukemia: Experience From a Tertiary Care Cancer Center of a Developing Country. J Pediatr Hematol Oncol. 2015;37: e143–9.
66. Hadley GP, Mars M, Ramdial PK. Bilateral Wilms’ tumour in a developing country: a descriptive study. Pediatr Surg Int. 2013;29: 419–423.
67. Hamid SA, Zia N, Maqsood S, Rafiq N, Fatima M, Syed Y, et al. Impact of dedicated pediatric neuro-oncological services in a developing country: A single-institution, Pakistani experience. Pediatr Blood Cancer. 2022;69: e29887.
68. Handayani K, Indraswari BW, Sitaresmi MN, Mulatsih S, Widjajanto PH, Kors WA, et al. Treatment Outcome of Children with Retinoblastoma in a Tertiary Care Referral Hospital in Indonesia. Asian Pac J Cancer Prev. 2021;22: 1613–1621.
69. Hazarika M, Sutnga C, Raj N, Roy PS, Sarangi SS, Reddy R, et al. Pediatric Hodgkin’s lymphoma: experience from a tertiary cancer center in North East India. Int J Contemp Pediatr. 2023;10: 204–210.
70. Herrera Toro N, Peña Aguirre L, Molina CF. Factores asociados a la sobrevida en pacientes con tumor de Wilms. Rev Colomb Cir. 2020;35: 621–629.
71. Herrera-Toro N, Peña-Aguirre L, Arango-Rave ME. Tumor de Wilms: experiencia de 12 años en dos hospitales de alto nivel en Medellín, Colombia. Iatreia. 2019;32: 82–91.
72. Hessissen L, Khtar R, Madani A, El Kababri M, Kili A, Harif M, et al. Improving the prognosis of pediatric Hodgkin lymphoma in developing countries: a Moroccan Society of Pediatric Hematology and Oncology study. Pediatr Blood Cancer. 2013;60: 1464–1469.
73. Holmes D, Matitiyo A, Mpasa A, Huibers M, Manda G, Tomoka T, et al. Outcomes of a resource-adapted Wilms tumor treatment protocol in Lilongwe, Malawi, 2016-2021: successes and enduring barriers to cure. bioRxiv. 2022. doi:10.1101/2022.08.08.22278537
74. Holmes DM, Matatiyo A, Mpasa A, Huibers MHW, Manda G, Tomoka T, et al. Outcomes of Wilms tumor therapy in Lilongwe, Malawi, 2016-2021: Successes and ongoing research priorities. Pediatr Blood Cancer. 2023;70: e30242.
75. Horn PRCB, Ribeiro-Carvalho M de M, Azevedo AMB de, Sousa AM de, Faria S, Wiggers C, et al. Long-Term Outcomes of Childhood Acute Lymphocytic Leukemia Treated with Adapted Berlin-Frankfurt-Münster (BFM) Protocols: A Multicentric Analysis from a Developing Country. Cancers (Basel). 2024;16. doi:10.3390/cancers16162898
76. Impact of using combined chemotherapy regimen in children with Hodgkin lymphoma in countries with limited resources: a single center experience in Iraq. Journal of Population Therapeutics and Clinical Pharmacology. 2023;30. doi:10.47750/jptcp.2023.30.03.067
77. Isa O N, Reyes C M, Russo N M. Resultado del tratamiento del tumor de Wilms en población pediátrica. Rev Chil Pediatr. 2013;84: 628–633.
78. Jabeen K, Ashraf MS, Iftikhar S, Belgaumi AF. The Impact of Socioeconomic Factors on the Outcome of Childhood Acute Lymphoblastic Leukemia (ALL) Treatment in a Low/Middle Income Country (LMIC). J Pediatr Hematol Oncol. 2016;38: 587–596.
79. Jaime-Pérez JC, Fernández LT, Jiménez-Castillo RA, Gómez-De León A, Cantú-Rodríguez OG, Gutiérrez-Aguirre CH, et al. Age Acts as an Adverse Independent Variable for Survival in Acute Lymphoblastic Leukemia: Data From a Cohort in Northeast Mexico. Clin Lymphoma Myeloma Leuk. 2017;17: 590–594.
80. Jaime-Pérez JC, Jiménez-Castillo RA, Pinzón-Uresti MA, Cantú-Rodríguez OG, Herrera-Garza JL, Marfil-Rivera LJ, et al. Real-world outcomes of treatment for acute lymphoblastic leukemia during adolescence in a financially restricted environment: Results at a single center in Latin America. Pediatr Blood Cancer. 2017;64. doi:10.1002/pbc.26396
81. Jaime-Pérez JC, López-Razo ON, García-Arellano G, Pinzón-Uresti MA, Jiménez-Castillo RA, González-Llano O, et al. Results of Treating Childhood Acute Lymphoblastic Leukemia in a Low-middle Income Country: 10 Year Experience in Northeast Mexico. Arch Med Res. 2016;47: 668–676.
82. Jaime-Pérez JC, Pinzón-Uresti MA, Jiménez-Castillo RA, Colunga-Pedraza JE, González-Llano Ó, Gómez-Almaguer D. Relapse of childhood acute lymphoblastic leukemia and outcomes at a reference center in Latin America: organomegaly at diagnosis is a significant clinical predictor. Hematology. 2018;23: 1–9.
83. Jain S, Kapoor G, Bajpai R. ABVD-Based Therapy for Hodgkin Lymphoma in Children and Adolescents: Lessons Learnt in a Tertiary Care Oncology Center in a Developing Country. Pediatr Blood Cancer. 2016;63: 1024–1030.
84. Jauquier N, English N, Davidson A, Cox SG. Solid malignancies during the first year of life: A 20-year review at Red Cross War Memorial Children’s Hospital, Cape Town, South Africa. S Afr Med J. 2022;112: 418–425.
85. Joseph LL, Boddu D, Srinivasan HN, Regi SS, Antonisamy B, John R, et al. Postchemotherapy tumor volume as a prognostic indicator in Wilms tumor: A single-center experience from South India. Pediatr Blood Cancer. 2022;69: e29454.
86. Kalinaki A, Muwonge H, Balagadde-Kambugu J, Mulumba Y, Ntende J, Ssali G, et al. Clinical presentation and outcomes in children with retinoblastoma managed at the Uganda Cancer Institute. J Cancer Epidemiol. 2022;2022: 8817215.
87. Karhan AN, Varan A, Akyüz C, Aydın B, Yalçın B, Kutluk T, et al. Outcome of 102 patients under 5 years of age with Hodgkin lymphoma. Arch Argent Pediatr. 2019;117: e459–e465.
88. Kersten E, Scanlan P, Dubois SG, Matthay KK. Current treatment and outcome for childhood acute leukemia in Tanzania. Pediatr Blood Cancer. 2013;60: 2047–2053.
89. Khan MS, Maaz AUR, Qazi AQ, Aslam S, Riaz S, Malik AS, et al. Prognostic impact of pre-referral tumor resection in unilateral Wilms tumor: A single-institute experience from a lower middle-income country. Pediatr Blood Cancer. 2024;71: e30760.
90. Khandelwal V, Choudhary D, Sharma SK, Doval D, Handoo A, Dadu T, et al. Treatment of newly diagnosed paediatric acute lymphoblastic leukaemia on BFM-based chemotherapy protocol: Feasibility and outcome from a tertiary care center in India. Pediatr Hematol Oncol J. 2023;8: 218–223.
91. Koka A, Saygin C, Uzunaslan D, Ozdemir N, Apak H, Celkan T. A 17-year experience with ALL-BFM protocol in acute lymphoblastic leukemia: prognostic predictors and interruptions during protocol. Leuk Res. 2014;38: 699–705.
92. Kriel M, Davidson A, Pillay K, Hendricks M, Phillips L-A. Clinicopathologic Characterization of Children With B-Cell Non-Hodgkin Lymphoma Over 10 Years at a Tertiary Center in Cape Town, South Africa. J Pediatr Hematol Oncol. 2020;42: e219–e227.
93. Kruger M, Reynders D, Omar F, Schoeman J, Wedi O, Harvey J. Retinoblastoma outcome at a single institution in South Africa. S Afr Med J. 2014;104: 859–863.
94. Kruger M, van Elsland SL, Afungchwi GM, Bardin R, Njodzeka B, Kouya F, et al. Outcome of retinoblastoma treatment protocol in Cameroon as per SIOP-PODC recommendation for a low-income setting. Pediatr Blood Cancer. 2022;69: e29642.
95. Kruger M, van Elsland SL, Davidson A, Stones D, du Plessis J, Naidu G, et al. Outcome of Retinoblastoma After Implementation of National Retinoblastoma Treatment Guidelines in South Africa. JCO Glob Oncol. 2024;10: e2400034.
96. Küpfer L, Meng B, Laurent D, Zimmermann M, Niggli F, Bourquin J-P, et al. Treatment of children with acute lymphoblastic leukemia in Cambodia. Pediatr Blood Cancer. 2021;68: e29184.
97. Kuyven NG de A. Estudo de Pacientes Primariamente Enucleados por Retinoblastoma Unilateral Intraocular Avançado, no Instituto Nacional de Câncer- Rio de Janeiro, no período de 1997 - 2015: Revisão Histopatológica e Fatores Associados ao Prognóstico. 2017 [cited 23 May 2025]. Available: http://docs.bvsalud.org/biblioref/coleciona-sus/2017/36461/36461-1660.pdf
98. Lashkari HP, Faheem M, Sridevi Hanaganahalli B, Bhat KG, Joshi J, Kamath N, et al. Resource limited centres can deliver treatment for children with acute lymphoblastic leukaemia with risk-stratified minimal residual disease based UKALL 2003 protocol with no modification and a good outcome. Expert Rev Hematol. 2020;13: 1143–1151.
99. Lepe-Zúñiga JL, Méndez-Cigarroa AO, Jerónimo-López FJ, Hernández-Orantes JG. Sobrevida global de pacientes con leucemia aguda en el Hospital de Especialidades Pediátricas de Chiapas, México. Boletín médico del Hospital Infantil de México. 2018;75: 338–351.
100. Lima ER, Resende JA, Ibiapina C da C, Oliveira BM de. Survival analysis of patients with central nervous tumor. Rev Médica Minas Gerais. 2015;25. doi:10.5935/2238-3182.20150091
101. Lopera Marín J, Suárez Mattos A, Gamboa O, Piña Quintero M, Terselich G. Sustitución de procarbazina por etopósido en un nuevo esquema de tratamiento OEPA/COPE en los niños y adolescentes con linfoma de Hodgkin: una evaluación de los resultados en el Instituto Nacional de Cancerología. Rev Colomb Cancerol. 2015;19: 222–228.
102. Luna-Fineman S, Castellanos M, Metzger ML, Baez LF, Peña Hernandez A, Bonilla M, et al. Treatment of high-risk Hodgkin lymphoma with a modified Stanford V regimen in the AHOPCA: Substituting chemotherapy agents and hampered outcomes. Pediatr Blood Cancer. 2024;71: e30792.
103. Machín García SA, Leblanch Fernández CC, García Caraballoso MB, Escalona Vives Y, Álvarez Molina I, Plá Del Toro MJ, et al. Caracterización de las leucemias en niños en Cuba (2006-2015). Rev Cubana Hematol Inmunol Hemoter. 2020;36. Available: http://scielo.sld.cu/scielo.php?script=sci_abstract&pid=S0864-02892020000100005&lng=es&nrm=iso&tlng=es
104. Madney Y, Abdalla A, Ahmed S, Romeih M, Fikry S, Mohammed E, et al. Exploring the complexity, treatment challenges, and outcomes in pediatric nodular lymphocyte predominant Hodgkin lymphoma: a perspective from a low-middle-income country. Front Oncol. 2024;14: 1432650.
105. Maher K MM, Mufeed K. HR, Salma S. H. Wilms’ tumor in children : A single institution 10-year experience. Egypt J Hosp Med. 2014;55: 159–164.
106. Makkeyah S, Manzour A, Tantawy A, Mohamed A, Ebeid F, El-Sherif N, et al. Treatment outcomes for childhood acute lymphoblastic leukemia in low-middle income country before minimal residual disease risk stratification. Cancer Epidemiol. 2021;75: 102040.
107. Malabanan-Cabebe CGP, Santos-Gonzales MA, Te AVR, Tan RJD, Gonzales-Sy J. Retinoblastoma in the Southern Philippines: Clinical Outcomes of Retinoblastoma Patients in a Davao Tertiary Hospital. Acta Med Philipp. 2024;58: 45–51.
108. Martijn HA, Njuguna F, Olbara G, Langat S, Skiles J, Martin S, et al. Influence of health insurance status on paediatric non-Hodgkin’s lymphoma treatment in Kenya. BMJ Paediatr Open. 2017;1: e000149.
109. Mattosinho C, Moura AT, Grigorovski N, Araújo LH, Ferman S, Ribeiro K. Socioeconomic status and retinoblastoma survival: Experience of a tertiary cancer center in Brazil. Pediatr Blood Cancer. 2021;68: e28757.
110. McGoldrick SM, Mutyaba I, Adams SV, Larsen A, Krantz EM, Namirembe C, et al. Survival of children with endemic Burkitt lymphoma in a prospective clinical care project in Uganda. Pediatr Blood Cancer. 2019;66: e27813.
111. Meena JP, Gupta AK, Parihar M, Seth R. Clinical profile and outcomes of Non-Hodgkin’s lymphoma in children: A report from a tertiary care hospital from India. Indian J Med Paediatr Oncol. 2019;40: 41–47.
112. Mehreen A, Wali RM, Sindhu II, Asad M, Ria S. Retrospective analysis of clinical features and treatment outcomes of children with Hodgkin’s Lymphoma treated with different chemotherapy protocols at a tertiary care center in Pakistan. J Pak Med Assoc. 2019;69: 1266–1272.
113. Melo A A, Artigas A CG, Muñoz N S, Brebi M P, Hoffstetter G R, Roa S JC. Perfil de Metilación de Genes Supresores de Tumores APAF1, ASSP1, p73 y FHIT en Pacientes con Leucemia Linfoblástica Aguda Infantil. Int J Morphol. 2013;31: 973–979.
114. Moleti ML, Al-Jadiry MF, Shateh WA, Al-Darraji AF, Mohamed S, Uccini S, et al. Long-term results with the adapted LMB 96 protocol in children with B-cell non Hodgkin lymphoma treated in Iraq: comparison in two subsequent cohorts of patients. Leuk Lymphoma. 2019;60: 1224–1233.
115. Moreira DC, González-Ramella O, Echavarría Valenzuela M, Carrillo AK, Faughnan L, Job G, et al. Evaluation of factors leading to poor outcomes for pediatric acute lymphoblastic leukemia in Mexico: a multi-institutional report of 2,116 patients. Front Oncol. 2023;13: 1255555.
116. Morosini F, Silveira A, Arias V, Castillo L. Incidence, mortality and survival of pediatric cancer in Uruguay 2011-2015. Rev Bras Cancerol. 2023;69. doi:10.32635/2176-9745.rbc.2023v69n1.3054
117. Moussa H, Sidhom I. NKX2-5, SIL/TAL and TLX3/HOX11L2 expression in Egyptian pediatric T-cell acute lymphoblastic leukemia. Asia Pac J Clin Oncol. 2016;12: e1–10.
118. Mutyaba I, Wabinga HR, Orem J, Casper C, Phipps W. Presentation and Outcomes of Childhood Cancer Patients at Uganda Cancer Institute. Glob Pediatr Health. 2019;6: 2333794X19849749.
119. Muulu MZ, Bvulani B, Shinondo P, Kaonga P. Factors Associated with Outcomes at 1 Year in Paediatric Post-nephrectomy Patients for Nephroblastoma at the University Teaching Hospital and Cancer Diseases Hospital in Lusaka, Zambia. Afr J Paediatr Surg. 2024;21: 172–177.
120. Nasir AA, Abdur-Raheem NT, Abdur-Rahman LO, Ibiyeye TT, Sayomi TO, Adedoyin OT, et al. Characteristics and Clinical Outcomes of Children With Wilms’ Tumour: A 15-year Experience in a Single Centre in Nigeria. J Pediatr Surg. 2024;59: 1009–1014.
121. Nina G. NM, Sardinas C. S. Cuadernos Hospital de Clínicas. Cuadernos Hospital de Clínicas. : 17.
122. Nindyastuti H, Rusmawatiningtyas D, Makrufardi F, Supriyadi E. Lag time to diagnosis as a predictor of mortality in children with extraocular retinoblastoma: Experience from a developing country. Asia Pac J Clin Oncol. 2022;18: 706–713.
123. Nishath T, Stacey AW, Steinberg D, Foster A, Bowman R, Essuman V, et al. Retinoblastoma survival and enucleation outcomes in 41 countries from the African continent. Br J Ophthalmol. 2024;109: 64–69.
124. Olbara G, van der Wijk T, Njuguna F, Langat S, Mwangi H, Skiles J, et al. Childhood acute lymphoblastic leukemia treatment in an academic hospital in Kenya: Treatment outcomes and health-care providers’ perspectives. Pediatr Blood Cancer. 2021;68: e29366.
125. Oliveira MCL, Sampaio KC, Brito AC, Campos MK, Murao M, Gusmão R, et al. 30 Years of Experience with Non-Hodgkin Lymphoma in Children and Adolescents: a retrospective cohort study. Rev Assoc Med Bras (1992). 2020;66: 25–30.
126. Paintsil V, David H, Kambugu J, Renner L, Kouya F, Eden T, et al. The Collaborative Wilms Tumour Africa Project; baseline evaluation of Wilms tumour treatment and outcome in eight institutes in sub-Saharan Africa. Eur J Cancer. 2015;51: 84–91.
127. Pan C, Cai J-Y, Xu M, Ye Q-D, Zhou M, Yin M-Z, et al. Renal tumor in developing countries: 142 cases from a single institution at Shanghai, China. World J Pediatr. 2015;11: 326–330.
128. Pant G, Verma N, Kumar A, Pooniya V, Gupta SK. Outcome of extraocular retinoblastoma in a resource limited center from low middle income country. Pediatr Hematol Oncol. 2017;34: 419–424.
129. Parkin DM, Youlden DR, Chitsike I, Chokunonga E, Couitchéré L, Gnahatin F, et al. Stage at diagnosis and survival by stage for the leading childhood cancers in three populations of sub-Saharan Africa. Int J Cancer. 2021;148: 2685–2691.
130. Pascual Morales C, Vasquez Ponce L, Hernandez Briceño J, Leon Lopez E, Guevara Guevara J, Jimenez Vargas J, et al. Clinical Factors, Management, and Outcomes of Patients Under 18 Years Old With Central Nervous System Tumors: Single-center Experience in Peru. J Pediatr Hematol Oncol. 2023;45: e345–e349.
131. Paula FDF, Elói-Santos SM, Xavier SG, Ganazza MA, Jotta PY, Yunes JA, et al. Comparison between qualitative and real-time polymerase chain reaction to evaluate minimal residual disease in children with acute lymphoblastic leukemia. Rev Bras Hematol Hemoter. 2015;37: 373–380.
132. Pérez V, Sampor C, Rey G, Parareda-Salles A, Kopp K, Dabezies AP, et al. Treatment of Nonmetastatic Unilateral Retinoblastoma in Children. JAMA Ophthalmol. 2018;136: 747–752.
133. Pérez-Cuevas R, Doubova SV, Zapata-Tarres M, Flores-Hernández S, Frazier L, Rodríguez-Galindo C, et al. Scaling up cancer care for children without medical insurance in developing countries: The case of Mexico. Pediatr Blood Cancer. 2013;60: 196–203.
134. Pongtanakul B, Sirachainan N, Surapolchai P, Charoenkwan P, Choeyprasert W, Komwilaisak P, et al. Pediatric primary central nervous system tumors registry in Thailand under National Health Security Office schemes. J Neurooncol. 2020;149: 141–151.
135. Pribnow AK, Ortiz R, Báez LF, Mendieta L, Luna-Fineman S. Effects of malnutrition on treatment-related morbidity and survival of children with cancer in Nicaragua. Pediatr Blood Cancer. 2017;64. doi:10.1002/pbc.26590
136. Qureshi SS, Bhagat MG, Kembhavi SA, Chinnaswamy G, Vora T, Prasad M, et al. A cross-sectional study of the distribution of pediatric solid tumors at an Indian tertiary cancer center. Indian J Cancer. 2018;55: 55–60.
137. Radhakrishnan V, Kritthivasan V, Kothandan BT, Srinivasan P, Das G, Ramamurthy J. Reducing chemotherapy dose intensity by 25% and adding rituximab improves survival in pediatric mature B-cell non-Hodgkin lymphoma in LMIC setting. Pediatr Blood Cancer. 2023;70: e30694.
138. Rahiman EA, Trehan A, Jain R, Menon P, Kakkar N, Srinivasan R, et al. A higher tumor volume and undernutrition at diagnosis adversely affect the survival of children with Wilms tumor: A study of 200 patients. Pediatr Blood Cancer. 2022;69: e29880.
139. Ramadhan MH, Sari NM, Peryoga SU, Susanah S. Survival and Treatment Outcomes of Childhood Acute Lymphoblastic Leukemia in a Low-Middle Income Country: A Single-Center Experience in West Java, Indonesia. J Blood Med. 2024;15: 77–85.
140. Ramirez O, Piedrahita V, Ardila J, Pardo C, Cabrera-Bernal E, Lopera J, et al. Primary central nervous system tumors survival in children in ten Colombian cities: a VIGICANCER report. Front Oncol. 2023;13: 1326788.
141. Reed JD, Masenge A, Buchner A, Omar F, Reynders D, Vorster M, et al. The Utility of Metabolic Parameters on Baseline F-18 FDG PET/CT in Predicting Treatment Response and Survival in Paediatric and Adolescent Hodgkin Lymphoma. J Clin Med. 2021;10. doi:10.3390/jcm10245979
142. Ribeiro KB, Veiga LHS, Carvalho Filho NP, Morton LM, Kleinerman RA, Antoneli CBG. Overall survival and cause-specific mortality in a hospital-based cohort of retinoblastoma patients in São Paulo, Brazil. Int J Cancer. 2025;156: 69–78.
143. Rojanaporn D, Chanthanaphak E, Boonyaopas R, Sujirakul T, Hongeng S, Ayudhaya SSN. Intra-Arterial Chemotherapy for Retinoblastoma: 8-Year Experience from a Tertiary Referral Institute in Thailand. Asia Pac J Ophthalmol (Phila). 2019;8: 211–217.
144. Rubagumya F, Xu MJ, May L, Driscoll C, Uwizeye FR, Shyirambere C, et al. Outcomes of Low-Intensity Treatment of Acute Lymphoblastic Leukemia at Butaro Cancer Center of Excellence in Rwanda. J Glob Oncol. 2018;4: 1–11.
145. San Roman M, Aguilo F, Clapes M, Sheku M, Dawoh P, Mora J, et al. Burkitt’s lymphoma treatment in a rural hospital in Sierra Leone. Trans R Soc Trop Med Hyg. 2013;107: 653–659.
146. Sankara P, Djiguimde WP, Ahnoux-Zabsonre A, Sanou J, Meda-Hien G, Diomande IA, et al. [Epidemio-clinical features of retinoblastoma at the Yalgado Ouedraogo University Hospital Center in Burkina Faso: about 32 cases]. Pan Afr Med J. 2020;37: 269.
147. Sarmiento-Urbina IC, Linares-Ballesteros A, Contreras-Acosta A, Cabrera-Bernal EV, Pardo-González CA, Uribe-Botero GI, et al. Resultados del Protocolo ACHOP 2006 en los niños con leucemia linfoblástica aguda en la Fundación HOMI Hospital de la Misericordia de Bogotá, en el periodo 2007 - 2012. Iatreia. 2019;32: 71–81.
148. Saxton AT, Bhattacharya M, Sivaraj D, Rice HE, Masalu N, Chao NJ, et al. Assessing the cost and economic impact of tertiary-level pediatric cancer care in Tanzania. PLoS One. 2022;17: e0273296.
149. Sayed DM, Sayed HAR, Raslan HN, Ali AM, Zahran A, Al-Hayek R, et al. Outcome and Clinical Significance of Immunophenotypic Markers Expressed in Different Treatment Protocols of Pediatric Patients With T-ALL in Developing Countries. Clin Lymphoma Myeloma Leuk. 2017;17: 443–449.
150. Seminara C, Planells MC, Pogonza RE, Morales M, Colaboradores. Wilms tumor: 15 years of experience at a children’s hospital in Córdoba, Argentina. Arch Argent Pediatr. 2019;117: 263–270.
151. Sevilla-Castillo RA, Andrade-Sarmiento LA. Factores asociados con supervivencia a cinco años en niños con astrocitoma cerebral. Gac Med Mex. 2018;154: 283–286.
152. Shah PK, Narendran V, Kalpana N. Outcomes of Intra- and Extraocular Retinoblastomas from a Single Institute in South India. Ophthalmic Genet. 2015;36: 248–250.
153. Shaheen N, Inayat N, Bashir S, Sheikh UN, Bakar MA, Rehman P. Survival outcomes of unilateral retinoblastoma based on pathological risk stratification-experience at a tertiary care centre in Pakistan. Ecancermedicalscience. 2022;16: 1360.
154. Shamoon RP, Ali MD, Shabila NP. Overview and outcome of Hodgkin’s Lymphoma: Experience of a single developing country's oncology centre. PLoS One. 2018;13: e0195629.
155. Sherief LM, Elsafy UR, Abdelkhalek ER, Kamal NM, Elbehedy R, Hassan TH, et al. Hodgkin lymphoma in childhood: clinicopathological features and therapy outcome at 2 centers from a developing country. Medicine (Baltimore). 2015;94: e670.
156. Shyirambere C, Villaverde C, Nguyen C, Ruhangaza D, Umwizerwa A, Nsanzimana O, et al. Nephroblastoma Treatment and Outcomes in a Low-Income Setting. JCO Glob Oncol. 2022;8: e2200036.
157. Silva KA de S, Rechenmacher C, Morais RV de, Michalowski MB, Daudt LE. Are there regional variations in the presentation of childhood leukemia? Clin Biomed Res. 2021. doi:10.22491/2357-9730.108722
158. Silveira HB, da Silveira APSF, Barcelos CC, Albuquerque JMT, Guerra RFO, Silva CTX. Perfil Clínico-Epidemiológico e Sobrevida dos Casos de Retinoblastoma em um Hospital Referência em Oncologia do Estado de Goiás. Rev Bras Cancerol. 2023;69: 243894.
159. Slone JS, Slone AK, Wally O, Semetsa P, Raletshegwana M, Alisanski S, et al. Establishing a Pediatric Hematology-Oncology Program in Botswana. J Glob Oncol. 2018;4: 1–9.
160. Smith ER, Cotache-Condor C, Leraas H, Truche P, Ward ZJ, Stefan C, et al. Towards attainment of the 2030 goal for childhood cancer survival for the World Health Organization Global Initiative for Childhood Cancer: An ecological, cross-sectional study. PLOS Glob Public Health. 2024;4: e0002530.
161. Soliman R, Bolous N, Heneghan C, Oke J, Boylan A-M, Eweida W, et al. An overview of childhood cancer care and outcomes in Egypt: a narrative review. Ecancermedicalscience. 2024;18: 1676.
162. Soliman RM, Elhaddad A, Oke J, Eweida W, Sidhom I, Ahmed S, et al. Temporal trends in childhood cancer survival in Egypt, 2007 to 2017: A large retrospective study of 14 808 children with cancer from the Children’s Cancer Hospital Egypt. Int J Cancer. 2021;148: 1562–1574.
163. Srinivasan S, Roy Moulik N, Kc A, Narula G, Sankaran H, Prasad M, et al. Increased toxicities in children with Burkitt lymphoma treated with rituximab: Experience from a tertiary cancer center in India. Pediatr Blood Cancer. 2020;67: e28682.
164. Stagno V, Mugamba J, Ssenyonga P, Kaaya BN, Warf BC. Presentation, pathology, and treatment outcome of brain tumors in 172 consecutive children at CURE Children’s Hospital of Uganda. The predominance of the visible diagnosis and the uncertainties of epidemiology in sub-Saharan Africa. Childs Nerv Syst. 2014;30: 137–146.
165. Stanley CC, Westmoreland KD, Heimlich BJ, El-Mallawany NK, Wasswa P, Mtete I, et al. Outcomes for paediatric Burkitt lymphoma treated with anthracycline-based therapy in Malawi. Br J Haematol. 2016;173: 705–712.
166. Stanley CC, Westmoreland KD, Itimu S, Salima A, van der Gronde T, Wasswa P, et al. Quantifying bias in survival estimates resulting from loss to follow-up among children with lymphoma in Malawi. Pediatr Blood Cancer. 2017;64. doi:10.1002/pbc.26370
167. Starling MTM, Pereira AAL, Arruda GV, Paiva WS, Neville IS, Restin FCF, et al. Brazilian pediatric patients with gliomas: treatment characteristics and survival outcomes. Rep Pract Oncol Radiother. 2024;29: 90–96.
168. Stary J, Zimmermann M, Campbell M, Castillo L, Dibar E, Donska S, et al. Intensive chemotherapy for childhood acute lymphoblastic leukemia: results of the randomized intercontinental trial ALL IC-BFM 2002. J Clin Oncol. 2014;32: 174–184.
169. Stefan DC, Lutchman R. Burkitt lymphoma: epidemiological features and survival in a South African centre. Infect Agent Cancer. 2014;9: 19.
170. Stones DK, De Bruin GP, Esterhuizen TM, Stefan DC. Childhood cancer survival rates in two South African units. S Afr Med J. 2014;104: 501–504.
171. Stulac S, Mark Munyaneza RB, Chai J, Bigirimana JB, Nyishime M, Tapela N, et al. Initiating Childhood Cancer Treatment in Rural Rwanda: A Partnership-Based Approach. Pediatr Blood Cancer. 2016;63: 813–817.
172. Suarez A, Piña M, Nichols-Vinueza DX, Lopera J, Rengifo L, Mesa M, et al. A strategy to improve treatment-related mortality and abandonment of therapy for childhood ALL in a developing country reveals the impact of treatment delays. Pediatr Blood Cancer. 2015;62: 1395–1402.
173. Tomar AS, Finger PT, Gallie B, Kivelä TT, Mallipatna A, Zhang C, et al. Global Retinoblastoma Treatment Outcomes: Association with National Income Level. Ophthalmology. 2021;128: 740–753.
174. Totadri S, Trehan A, Kaur A, Bansal D. Effect of socio-economic status & proximity of patient residence to hospital on survival in childhood acute lymphoblastic leukaemia. Indian J Med Res. 2019;149: 26–33.
175. Trabelsi S, Brahim DH-B, Ladib M, Mama N, Harrabi I, Tlili K, et al. Glioma epidemiology in the central Tunisian population: 1993-2012. Asian Pac J Cancer Prev. 2014;15: 8753–8757.
176. Traoré F, Sylla F, Togo B, Kamaté B, Diabaté K, Diakité AA, et al. Treatment of retinoblastoma in Sub-Saharan Africa: Experience of the paediatric oncology unit at Gabriel Toure Teaching Hospital and the Institute of African Tropical Ophthalmology, Bamako, Mali. Pediatr Blood Cancer. 2018;65: e27101.
177. Trehan A, Bansal D, Varma N, Vora A. Improving outcome of acute lymphoblastic leukemia with a simplified protocol: report from a tertiary care center in north India. Pediatr Blood Cancer. 2017;64. doi:10.1002/pbc.26281
178. Trehan A, Singla S, Marwaha RK, Bansal D, Srinivasan R. Hodgkin lymphoma in children: experience in a tertiary care centre in India. J Pediatr Hematol Oncol. 2013;35: 174–179.
179. Trujillo AM, Linares Ballesteros A, Sarmiento IC. Intensive chemotherapy in children with acute lymphoblastic leukemia. Interim analysis in a referral center in Colombia. Rev Fac Med Univ Nac Colomb. 2016;64: 417.
180. Uche EO, Eke CB, Okafor OC, Uche NJ, Ajuzieogu OV, Amuta DS, et al. Pediatric brain tumor care in a Sub-Saharan setting: current poise of a precariously loaded dice. Br J Neurosurg. 2021;35: 174–180.
181. Uittenboogaard A, Njuguna F, Mostert S, Langat S, van de Velde ME, Olbara G, et al. Outcomes of Wilms tumor treatment in western Kenya. Pediatr Blood Cancer. 2022;69: e29503.
182. Utomo PT, Respatika D, Ardianto B, Rinonce HT, Heriyanto DS, Dibyasakti BA, et al. Lag time, high-risk histopathological features, metastasis, and survival interrelation in retinoblastoma: a perspective from lower-middle income country. Int J Ophthalmol. 2022;15: 1994–2000.
183. Varghese B, Joobomary AA, Savida P. Five-year survival rate and the factors for risk-directed therapy in acute lymphoblastic leukemia. Indian J Med Paediatr Oncol. 2018;39: 301–306.
184. Verma N, Kumar A. Clinicoepidemiological Profile and Outcome of Children With Wilms Tumor in a Developing Country. J Pediatr Hematol Oncol. 2016;38: e213–6.
185. Viana SS, de Lima LMMR, do Nascimento JB, Cardoso CAF, Rosário ACD, Mendonça C de Q, et al. Secular trends and predictors of mortality in acute lymphoblastic leukemia for children of low socioeconomic level in Northeast Brazil. Leuk Res. 2015;39: 1060–1065.
186. Villegas MG, Ruiz FE, Urdaneta N, Gutiérrez E, Gimón AV, Barboza D, et al. Tumores cerebrales pediátricos experiencia de 10 años. Revista Venezolana de Oncologia. 2013;25: 85–97.
187. Waddell K, Matua M, Bidwell C, Atwine R, Onyango J, Picton SV, et al. A ten-year study of Retinoblastoma in Uganda: An approach to improving outcome with limited resources. Cancer Epidemiol. 2021;71: 101777.
188. Ward R, Jones HM, Witt D, Boop F, Bouffet E, Rodriguez-Galindo C, et al. Outcomes of Children With Low-Grade Gliomas in Low- and Middle-Income Countries: A Systematic Review. JCO Glob Oncol. 2022;8: e2200199.
189. Wesevich A, Mocha G, Kiwara F, Chao C, Shabani I, Igenge JZ, et al. Wilms tumor treatment protocol compliance and the influence on outcomes for children in Tanzania. Pediatr Blood Cancer. 2023;70: e30704.
190. Westmoreland KD, Montgomery ND, Stanley CC, El-Mallawany NK, Wasswa P, van der Gronde T, et al. Plasma Epstein-Barr virus DNA for pediatric Burkitt lymphoma diagnosis, prognosis and response assessment in Malawi. Int J Cancer. 2017;140: 2509–2516.
191. Yao AJ-J, Moreira C, Traoré F, Kaboret S, Pondy A, Rakotomahefa Narison ML, et al. Treatment of Wilms Tumor in Sub-Saharan Africa: Results of the Second French African Pediatric Oncology Group Study. J Glob Oncol. 2019;5: 1–8.
192. Yilmaz B, Koc A, Dogru O, Tufan Tas B, Senay RE. The results of the modified St Jude Total Therapy XV Protocol in the treatment of low- and middle-income children with acute lymphoblastic leukemia. Leuk Lymphoma. 2023;64: 1304–1314.
193. Yizhuo W, Dongsheng H, Jitong S, Jianmin M, Junyang Z, Bin L, et al. Clinical treatment and prognostic observation for different pathological infiltrations in 537 patients with unilateral retinoblastoma. Chin Med J (Engl). 2014;127: 3581–3586.
194. Zahir M, Alidousti A, Kajbafzadeh A-M, Arshadi H, Kompani F, Hajivalizadeh S, et al. Current status and future perspectives of wilms tumor treatment in Iran. Ann Med Surg (Lond). 2023;85: 1425–1429.
195. Zaruma-Torres F, Lares-Asseff I, Reyes-Espinoza A, Lopera-Castañeda V, Almanza-Reyes H, Arias-Peláez MC. Impacto de polimorfismos genéticos de la vía metabólica del metotrexato sobre la sobrevida de niños mexicanos con leucemia linfoblástica aguda (LLA). Rev Vitae. 2015;22. doi:10.17533/udea.vitae.v22n3a02
196. Zouain-Figueiredo GP, Zandonade E, Amorim MHC. Cancer survival among children and adolescents at a state referral hospital in southeastern Brazil. Rev Bras Saude Mater Infant. 2013;13: 335–344.

**Table S7. Country representation by World Bank income classification**

| **Income Classification** | **Countries Represented** | **No. of Studies** | **Percentage of Total Studies** |
| --- | --- | --- | --- |
| Low-income | Botswana, Burkina Faso, Malawi, Mali, Rwanda, Sierra Leone, Sudan, Uganda | 23 | 11.7% |
| Lower-middle-income | Bolivia, Cambodia, Cameroon, Egypt, India, Kenya, Morocco, Nicaragua, Nigeria, Pakistan, Philippines, Tanzania, Venezuela, Zambia | 67 | 34.2% |
| Upper-middle-income | Argentina, Brazil, China, Colombia, Cuba, Ecuador, Indonesia, Iran, Iraq, Jordania, Mexico, Peru, South Africa, Thailand, Tunisia, Turkey | 82 | 41.8% |
| High-income (Global South) | Chile, Uruguay | 4 | 2.0% |
| Multicountry | Multiple countries | 20 | 10.0% |

**Table S8. Interactive evidence maps**

| Studies included | <https://flourish-user-preview.com/22542303/Kfjxt67XVnM-MTY5SAfLe8Vfwz5Pq2VY4DKBgZ43yc2A710JQXlOLe8zUFPI7zmO/> |
| --- | --- |
| ALL | <https://flourish-user-preview.com/22428179/_2Nlo3a5JHZgcpcGOnW_PVb-K1iL4Le94nMGaEmSXzOgPGu7Oa-zSBHiBrV_VnuN/> |
| Burkitt | <https://flourish-user-preview.com/22505606/F-7lFg2raESPoL6sBHR2nsSVrFaOPbdLBZYkN5caSfNLTN0U5ygqxTJplIYVqOCt/> |
| Hodking | <https://flourish-user-preview.com/22514516/TrGMunxcgPoC34UkfKMR-LLu-BCfip_7xi72prDyKra4H-2lCQ47s-dafpsieKd7/> |
| Low-grade glioma | <https://flourish-user-preview.com/22579038/cX_-64hhGtdNNZtB8IcV5jOc-Hm8jKmlyhrghs_S3fOQj9G80ECkN2_jAbQACMY5/> |
| Retinoblastoma | <https://flourish-user-preview.com/22604861/61CpygX9qaL8thq6nNWbr5i1jJdNTvDBM_7b7QmF_VRxqurNClfVTeIaSG-RVUdB/> |
| Wilms | <https://flourish-user-preview.com/22516611/H-yrpX0gCQGw1xtVgIy_Wjj1Z1S6z0G7JO7vjqqNnE-Jr2HKqkZ9dySSVb8O7JaR/> |

**Table S9. Quality assessment**

| **Author** | **Year** | **1. Time frame mentioned** | **2. Multi-institutional** | **3. Prospective** | **4. Inclusion criteria described** | **5. Study size >20** | **6. Treatment described** | **7. Patient Follow-up >2 years** | **8. EFS included** | **9. Survival is calculated** | **10. Mention of study limitations** |
| --- | --- | --- | --- | --- | --- | --- | --- | --- | --- | --- | --- |
| Gaytan-Morales | 2018 | low | low | high | low | low | low | low | low | low | low |
| Ghafoor | 2019 | low | high | high | low | low | low | low | low | low | unclear |
| Adegoke | 2024 | low | high | low | low | low | high | unclear | high | high | high |
| Ahmad | 2023 | low | high | low | low | low | low | low | high | low | high |
| Alakaloko | 2022 | low | high | high | low | low | low | low | high | low | high |
| Al-Hadad | 2021 | low | high | unclear | low | low | low | low | low | low | high |
| Al-Jumaily | 2024 | low | high | high | low | low | low | low | low | low | low |
| Alkayed | 2013 | low | high | high | unclear | low | low | low | low | low | low |
| Almasi-Hashiani | 2013 | low | high | high | low | low | high | low | high | low | high |
| Antonisamy | 2024 | low | high | high | low | high | high | low | low | low | high |
| Anwar | 2017 | low | high | high | unclear | low | unclear | high | high | unclear | low |
| Anyanwu | 2015 | low | high | high | low | low | low | unclear | high | high | high |
| Appeadu-Mensah | 2024 | low | low | low | low | low | low | high | low | high | low |
| Arazi | 2025 | low | low | low | low | low | unclear | low | high | low | high |
| Aristizabal | 2023 | low | high | low | low | low | low | low | low | low | low |
| Aronson | 2014 | low | high | high | low | high | high | low | low | low | high |
| Arora | 2023 | low | unclear | high | low | low | high | low | low | low | low |
| Asfour | 2020 | low | high | high | low | low | low | low | low | low | low |
| Assumpção | 2013 | unclear | low | low | high | low | high | unclear | low | low | high |
| Atima | 2023 | low | high | low | low | low | low | low | high | low | low |
| Bahoush | 2020 | low | high | high | low | low | low | low | low | low | low |
| Barragán-Pérez | 2020 | low | high | high | low | low | low | low | high | low | low |
| Belgaumi | 2016 | low | high | high | low | low | low | low | low | low | high |
| Berry | 2024 | low | low | low | low | low | low | low | high | low | low |
| Bordbar | 2023 | low | high | high | low | low | low | low | low | low | low |
| Bouda | 2019 | low | low | low | low | low | low | high | high | low | low |
| Brandalise | 2016 | low | low | low | low | low | low | low | low | low | low |
| Bravo | 2013 | low | low | low | low | low | high | low | high | low | low |
| Brito Silveira | 2023 | low | high | high | low | low | unclear | high | high | low | low |
| Buckle | 2016 | low | high | low | low | low | low | low | high | low | low |
| Bukhari | 2023 | low | high | high | low | low | low | low | low | low | low |
| Businge | 2024 | low | low | high | low | low | high | low | high | low | low |
| Carvalho | 2016 | low | high | high | unclear | low | high | low | low | low | high |
| Chagaluka | 2020 | low | low | low | low | low | low | low | low | high | unclear |
| Chapman | 2024 | low | high | high | high | low | low | high | low | low | high |
| Chauhan | 2021 | low | low | high | low | low | low | low | low | low | low |
| Chen | 2013 | low | high | high | low | low | low | low | low | low | high |
| Cueva-Arica | 2024 | low | high | high | low | low | low | low | low | low | low |
| Davidson | 2013 | low | high | high | low | low | low | low | low | low | low |
| de Souza Silva | 2021 | low | high | high | low | low | low | unclear | low | low | high |
| DeBoer | 2020 | low | high | high | low | low | low | low | high | low | low |
| Denburg | 2019 | low | high | low | low | low | low | high | high | low | low |
| Duffy | 2023 | low | low | high | low | low | low | low | low | low | low |
| Dufort | 2021 | low | low | unclear | low | low | unclear | low | high | low | low |
| Dujua | 2018 | low | low | high | low | low | low | low | low | low | high |
| Ekuk | 2023 | low | high | high | low | low | low | unclear | high | low | high |
| Elhassan | 2019 | low | high | high | low | low | high | unclear | high | low | low |
| El-Mallawany | 2017 | low | high | high | low | low | low | high | high | low | low |
| Elzomor | 2017 | low | high | high | unclear | low | low | low | high | low | low |
| Farrag | 2023 | low | high | high | low | low | high | unclear | low | low | high |
| Fawzy | 2018 | low | high | low | low | low | low | low | low | low | unclear |
| Fufa | 2024 | low | low | low | low | low | low | unclear | low | low | low |
| Geel | 2020 | low | low | high | low | low | low | low | high | low | low |
| Geel | 2023 | low | low | low | low | low | low | low | unclear | low | low |
| Geel | 2017 | low | low | high | low | low | low | low | high | low | low |
| Ghafoor | 2020 | low | high | high | low | low | low | unclear | low | low | low |
| Gibson | 2018 | low | low | high | low | low | unclear | high | low | low | low |
| Girardi | 2020 | low | low | high | low | low | high | unclear | unclear | unclear | low |
| Gonález-Otero | 2014 | low | low | unclear | unclear | low | low | high | low | low | high |
| Grace | 2024 | low | high | high | high | low | high | low | high | low | high |
| GRIGOROVSKI | 2017 | low | high | high | low | low | low | low | low | low | low |
| Guerrero | 2020 | low | high | high | low | low | low | low | low | low | high |
| Güneş | 2014 | low | low | high | low | low | low | low | low | low | low |
| Gupta | 2015 | low | high | high | low | low | low | low | low | low | low |
| Guzmán | 2017 | low | high | high | low | low | low | high | low | low | low |
| Hadley | 2013 | low | high | high | low | high | low | high | high | low | high |
| Hamid | 2022 | low | high | high | high | low | high | low | high | high | unclear |
| Handayani | 2021 | low | high | high | low | low | low | low | low | low | low |
| Hazarika | 2023 | low | high | high | low | low | low | low | low | low | low |
| Herrera-Toro | 2020 | low | low | high | low | low | low | unclear | high | unclear | low |
| Herrera-Toro | 2019 | low | low | high | low | low | low | low | low | low | low |
| Hessissen | 2013 | low | low | low | low | low | low | low | low | low | high |
| Holmes | 2023 | low | high | high | low | low | low | low | low | low | low |
| Holmes | 2022 | low | high | high | low | low | low | low | low | low | low |
| Horn | 2024 | low | low | high | low | low | low | low | low | low | low |
| Isa | 2013 | low | high | high | low | low | low | low | high | low | high |
| Jabeen | 2016 | low | high | high | low | low | low | low | low | low | low |
| Jaime-P erez | 2017 | low | high | high | low | low | low | low | low | low | low |
| Jaime-Pérez | 2018 | low | high | low | low | low | low | low | low | low | high |
| Jaime-Pérez | 2017 | low | high | high | high | low | high | low | low | low | high |
| Jain | 2016 | low | high | high | low | low | low | low | low | low | low |
| Jauquier | 2022 | low | high | high | low | low | high | low | high | low | low |
| Joseph | 2022 | low | high | high | low | low | low | low | low | low | low |
| Kalinaki | 2022 | low | high | low | low | low | high | low | high | low | high |
| Karhan | 2019 | low | high | low | low | low | low | low | low | low | high |
| Kersten | 2013 | low | high | high | low | low | low | low | low | high | low |
| Khandelwal | 2023 | low | high | high | low | low | low | low | low | low | low |
| Koka | 2014 | low | high | high | low | low | low | low | low | high | low |
| Kriel | 2019 | low | high | high | high | low | low | low | low | low | low |
| Kruger | 2024 | low | low | low | low | low | low | high | high | low | low |
| Kruger | 2014 | unclear | high | low | high | low | low | low | low | low | high |
| Kruger | 2022 | low | low | low | low | low | low | high | high | low | high |
| Küpfer | 2021 | low | high | high | low | low | low | low | unclear | low | high |
| Lashkari | 2020 | low | high | high | low | low | low | low | low | low | low |
| Lepe-Zúñiga | 2018 | low | high | high | low | low | high | low | high | low | high |
| Lopera Marín | 2015 | low | high | high | low | low | low | low | low | low | low |
| Luna-Fineman | 2023 | low | low | low | low | low | low | low | low | low | high |
| Machin Garcia | 2020 | low | low | high | low | low | high | low | low | low | high |
| Madney | 2024 | low | high | high | low | low | low | low | low | low | low |
| Maher | 2014 | low | high | high | low | low | unclear | low | low | low | low |
| Makkeyah | 2021 | low | high | high | low | low | low | low | low | low | low |
| Marín | 2015 | low | high | high | low | low | low | low | low | low | low |
| Martijn | 2017 | low | high | high | low | low | low | low | low | high | low |
| Mattosinho | 2020 | low | high | high | low | low | unclear | low | high | low | low |
| McGoldrick | 2019 | low | high | low | low | low | low | low | high | low | low |
| Mehreen | 2019 | low | high | high | low | low | low | low | low | low | high |
| Melo | 2013 | low | high | low | low | low | high | low | high | low | high |
| Moleti | 2019 | low | high | low | low | low | low | low | low | low | high |
| Monika Hasna Ramadhan | 2024 | low | high | high | low | low | high | high | low | low | low |
| Moreira | 2023 | low | low | high | low | low | low | low | low | low | low |
| Morosini | 2023 | low | low | high | low | low | high | low | high | low | high |
| Moussa | 2016 | low | high | low | high | low | low | high | low | low | high |
| Mutyaba | 2019 | low | high | high | low | low | high | high | high | low | low |
| Muulu | 2024 | low | high | high | low | low | low | high | low | low | high |
| Nasir | 2024 | low | high | high | low | low | low | unclear | high | unclear | unclear |
| Nina | 2018 | low | high | high | low | low | low | high | low | low | low |
| Nindyastuti | 2022 | low | high | high | low | low | low | low | high | low | low |
| Nishath | 2024 | low | low | low | low | low | unclear | low | high | low | low |
| Olbara | 2021 | low | high | high | low | low | low | low | low | high | low |
| Paintsil | 2015 | low | low | high | low | low | low | unclear | high | unclear | high |
| Pan | 2015 | low | high | high | low | low | low | low | low | low | low |
| Pant | 2024 | low | high | high | low | low | low | low | high | low | low |
| Parkin | 2020 | low | low | high | low | low | high | low | high | low | low |
| Pascual Morales | 2022 | low | high | high | low | low | high | low | high | low | low |
| Paula | 2015 | low | low | high | low | low | low | unclear | low | low | high |
| Pe ́rez-Cuevas | 2012 | low | low | high | low | low | high | low | high | low | low |
| Pérez | 2018 | low | high | low | low | low | low | low | low | low | low |
| Pongtanakul | 2020 | low | low | unclear | low | low | low | low | high | low | low |
| Prasad | 2021 | low | high | high | low | low | low | low | low | low | low |
| Pribnow | 2017 | low | high | high | low | low | high | unclear | low | high | low |
| Qureshi | 2018 | low | high | high | low | low | low | low | high | low | low |
| Radhakrishnan | 2023 | low | high | low | low | low | low | low | low | low | low |
| Rahiman | 2022 | low | high | high | low | low | low | low | low | low | high |
| Ramirez | 2024 | low | low | low | low | low | high | low | high | low | low |
| Rehman | 2020 | low | high | high | high | high | high | high | high | high | low |
| Ribeiro | 2024 | low | high | high | unclear | low | low | low | high | low | low |
| Ribeiro Lima | 2015 | low | high | high | low | low | high | low | high | low | low |
| Rodrigo Blanco | 2013 | low | high | high | high | low | high | low | high | low | high |
| Rojanaporn | 2019 | low | high | high | unclear | low | low | low | high | low | low |
| Rubagumya | 2018 | low | high | high | low | low | low | low | low | low | low |
| Saghir Khan | 2024 | low | high | high | low | low | low | low | low | low | high |
| San Roman | 2013 | low | high | low | low | low | low | high | high | low | high |
| Sankara | 2020 | low | high | high | low | low | low | unclear | high | low | low |
| Sarmiento-Urbina | 2018 | low | high | high | low | low | low | low | low | low | low |
| Saxton | 2022 | low | high | high | low | low | high | unclear | high | low | low |
| Sayed | 2017 | low | high | high | low | low | low | low | low | low | high |
| Seminara | 2019 | low | high | high | low | low | low | low | low | low | high |
| Sevilla-Castillo | 2017 | low | high | high | low | low | low | low | high | low | low |
| Shah | 2015 | low | high | high | low | low | low | low | high | low | high |
| Shaheen | 2021 | low | high | high | low | low | low | low | low | low | high |
| Shamoon | 2018 | low | high | high | low | low | low | low | low | low | high |
| Sherief | 2015 | low | low | high | low | low | low | low | low | low | high |
| Shyirambere | 2022 | low | high | high | low | low | low | unclear | high | low | low |
| Silva | 2016 | low | high | low | low | high | low | low | high | low | high |
| Silvia Regina Brandalise | 2016 | low | low | low | low | low | low | low | low | low | high |
| Slone | 2018 | low | high | high | low | low | high | high | high | low | low |
| Smith | 2024 | low | low | unclear | low | unclear | high | unclear | high | low | high |
| Soliman | 2024 | high | low | high | low | unclear | high | unclear | high | high | high |
| Srinivasan | 2020 | low | high | high | low | low | low | low | low | low | low |
| Stacey | 2024 | low | low | low | low | low | unclear | low | high | low | low |
| Stagno | 2013 | low | high | high | high | low | high | low | high | low | low |
| Stanley | 2016 | low | high | low | low | low | low | high | high | low | low |
| Stanley | 2016 | low | high | low | low | low | low | low | high | low | low |
| Starling | 2024 | low | low | high | low | low | low | low | high | low | low |
| Stary | 2014 | low | low | low | low | low | low | low | low | low | low |
| Stefan | 2014 | low | high | high | low | low | low | unclear | high | low | low |
| Stones | 2014 | low | low | high | low | low | high | high | high | low | low |
| Stulac | 2016 | low | high | high | low | low | low | unclear | unclear | unclear | low |
| Suarez | 2015 | low | high | low | low | low | low | low | low | low | low |
| Suzy Abdelmabood | 2020 | low | high | high | low | low | low | low | low | low | high |
| Swaminathan | 2008 | low | low | high | high | low | high | unclear | high | low | high |
| The global retinoblastoma study group | 2022 | low | low | low | low | low | high | low | high | low | low |
| Tomar | 2021 | low | low | high | high | low | high | low | high | low | low |
| Totadri | 2019 | low | high | high | low | low | low | low | low | low | high |
| Trabelsi | 2014 | low | low | high | low | low | high | high | high | low | high |
| Traoré | 2018 | low | high | low | low | low | high | high | low | low | high |
| Trehan | 2017 | low | high | high | high | low | low | low | low | low | low |
| Trehan | 2013 | low | high | high | low | low | low | low | low | low | high |
| Trujillo | 2016 | low | high | high | low | low | low | high | low | low | low |
| Uche | 2013 | low | high | high | low | high | high | low | high | low | high |
| Uche | 2021 | low | high | low | low | high | high | low | high | high | low |
| Uittenboogaard | 2021 | low | high | high | low | low | low | unclear | low | low | low |
| Utomo | 2022 | low | high | high | high | low | high | low | high | high | low |
| Varghese | 2018 | low | high | high | low | low | low | unclear | low | low | high |
| Verma | 2016 | low | high | high | low | low | low | low | low | low | high |
| Viana | 2015 | low | low | unclear | low | low | low | low | high | low | high |
| Villegas | 2013 | low | high | high | low | low | low | low | low | low | high |
| Waddell | 2021 | low | high | low | high | low | low | unclear | high | high | high |
| Wesevich | 2023 | low | high | high | low | low | low | low | low | low | low |
| Westmoreland | 2017 | low | high | low | low | low | high | low | high | low | low |
| Yao | 2019 | low | low | low | low | low | low | low | low | low | low |
| Yimaz | 2023 | low | high | high | low | high | low | low | low | low | high |
| Yizhuo | 2014 | low | high | high | high | low | low | low | high | low | high |
| Zahir | 2023 | low | high | high | low | low | low | low | high | low | low |
| Zaruma-Torres | 2015 | low | high | high | low | low | low | high | low | low | high |
| Zouain-Figueiredo | 2013 | low | high | high | low | low | low | low | high | low | low |

**Quality assessment: JBI Criteria**

The articles that the study design was not clarified are not included in this tables.

JBI criteria in cohort studies

| **Author** | **Year** | **Were the groups comparable at baseline?** | **Was the exposure/intervention clearly defined?** | **Was the exposure/intervention measured reliably?** | **Were the outcomes clearly defined?** | **Were confounding factors identified and managed?** | **Were the outcomes measured reliably?** | **Was follow-up time adequate? (2 years)** | **Were statistical analyses appropriate?** | **Overall appraisal** |
| --- | --- | --- | --- | --- | --- | --- | --- | --- | --- | --- |
| Shyirambere | 2022 | not applicable | yes | yes | yes | yes | yes | unclear | yes | low risk |
| Atima | 2023 | not applicable | yes | not applicable | yes | yes | yes | yes | yes | low risk |
| Asfour | 2020 | not applicable | yes | not applicable | yes | yes | yes | yes | yes | low risk |
| Saxton | 2022 | not applicable | no | no | yes | no | yes | unclear | unclear | high risk |
| Stacey | 2024 | not applicable | no | no | yes | unclear | yes | yes | yes | low risk |
| Barragán-Pérez | 2020 | not applicable | yes | yes | yes | yes | yes | yes | yes | low risk |
| Fawzy | 2018 | not applicable | yes | no | yes | yes | yes | yes | yes | low risk |
| Al-Hadad | 2021 | unclear | yes | yes | yes | yes | yes | yes | yes | low risk |
| Jaime-Pérez | 2018 | not applicable | yes | yes | yes | yes | yes | yes | yes | low risk |
| Sayed | 2017 | not applicable | yes | yes | yes | yes | yes | yes | yes | low risk |
| Trehan | 2017 | not applicable | yes | yes | yes | yes | yes | yes | yes | low risk |
| Kersten | 2013 | not applicable | yes | yes | yes | yes | yes | yes | yes | low risk |
| Jaime-Pérez | 2017 | not applicable | yes | yes | yes | yes | yes | yes | yes | low risk |
| Koka | 2014 | not applicable | yes | yes | yes | yes | yes | yes | yes | low risk |
| Gupta | 2015 | yes | yes | yes | yes | yes | yes | unclear | yes | low risk |
| Totadri | 2019 | not applicable | yes | yes | yes | yes | yes | yes | yes | low risk |
| Moussa | 2016 | not applicable | yes | yes | yes | unclear | yes | no | yes | unclear |
| Küpfer | 2021 | not applicable | yes | yes | yes | yes | yes | yes | yes | low risk |
| Ahmad | 2023 | not applicable | yes | yes | yes | yes | yes | yes | yes | low risk |
| Lashkari | 2020 | not applicable | yes | yes | yes | yes | yes | yes | yes | low risk |
| Aristizabal | 2023 | unclear | yes | yes | yes | no | yes | yes | yes | low risk |
| Rubagumya | 2018 | not applicable | yes | yes | yes | no | yes | yes | yes | low risk |
| Makkeyah | 2021 | not applicable | yes | yes | yes | yes | yes | yes | yes | low risk |
| Yimaz | 2023 | yes | yes | yes | yes | yes | yes | yes | yes | low risk |
| Duffy | 2023 | not applicable | yes | yes | yes | unclear | yes | yes | yes | low risk |
| Moreira | 2023 | not applicable | yes | yes | no | unclear | yes | yes | yes | low risk |
| Monika Hasna Ramadhan | 2024 | not applicable | yes | unclear | yes | unclear | unclear | unclear | yes | high risk |
| Suzy Abdelmabood | 2020 | not applicable | yes | yes | yes | unclear | yes | yes | yes | low risk |
| Almasi-Hashiani | 2013 | not applicable | yes | yes | yes | unclear | unclear | yes | yes | unclear |
| Assumpção | 2013 | unclear | yes | yes | yes | unclear | yes | unclear | yes | unclear |
| Carvalho | 2016 | no | yes | yes | yes | no | yes | unclear | unclear | high risk |
| Cueva-Arica | 2024 | yes | yes | yes | yes | no | yes | unclear | yes | unclear |
| Gonález-Otero | 2014 | not applicable | yes | yes | yes | no | yes | no | yes | unclear |
| Alkayed | 2013 | unclear | yes | yes | yes | no | yes | yes | yes | low risk |
| Melo | 2013 | unclear | not applicable | not applicable | yes | no | unclear | yes | yes | high risk |
| Nina | 2018 | not applicable | yes | yes | yes | no | yes | no | yes | low risk |
| Paula | 2015 | yes | yes | yes | yes | no | yes | unclear | yes | low risk |
| Trujillo | 2016 | yes | yes | yes | yes | no | yes | unclear | yes | low risk |
| Zaruma-Torres | 2015 | unclear | yes | yes | yes | no | yes | no | yes | unclear |
| Horn | 2024 | yes | yes | yes | yes | no | yes | yes | yes | low risk |
| Antonisamy | 2024 | yes | not applicable | not applicable | yes | no | yes | yes | yes | low risk |
| Khandelwal | 2023 | yes | not applicable | not applicable | yes | no | yes | yes | yes | low risk |
| Dujua | 2018 | not applicable | not applicable | yes | yes | no | yes | yes | yes | low risk |
| Jaime-Pérez | 2017 | no | yes | yes | yes | unclear | yes | yes | yes | unclear |
| Güneş | 2014 | not applicable | yes | yes | yes | yes | yes | yes | yes | low risk |
| Olbara | 2021 | not applicable | yes | yes | yes | yes | yes | yes | yes | low risk |
| Nishath | 2024 | not applicable | unclear | yes | yes | yes | yes | yes | yes | low risk |
| Mattosinho | 2020 | not applicable | unclear | no | yes | yes | yes | yes | yes | low risk |
| Berry | 2024 | not applicable | yes | yes | yes | yes | yes | yes | yes | low risk |
| Ribeiro | 2024 | not applicable | yes | yes | yes | yes | yes | yes | yes | low risk |
| Pant | 2024 | not applicable | yes | yes | yes | yes | yes | yes | yes | low risk |
| Nindyastuti | 2022 | not applicable | yes | yes | yes | yes | yes | yes | yes | low risk |
| Shah | 2015 | not applicable | yes | yes | unclear | unclear | unclear | yes | yes | unclear |
| Waddell | 2021 | not applicable | yes | yes | yes | unclear | no | no | yes | unclear |
| Tomar | 2021 | no | yes | yes | yes | unclear | yes | yes | yes | low risk |
| Traoré | 2018 | not applicable | yes | yes | yes | unclear | yes | yes | yes | low risk |
| Kruger | 2024 | not applicable | yes | yes | yes | unclear | yes | yes | yes | low risk |
| Kruger | 2014 | yes | yes | yes | yes | no | yes | yes | yes | low risk |
| Pérez | 2018 | not applicable | yes | yes | no | yes | yes | yes | yes | low risk |
| The global retinoblastoma study group | 2022 | no | yes | yes | yes | no | yes | yes | yes | low risk |
| Rehman | 2020 | not applicable | yes | no | yes | no | no | yes | yes | high risk |
| Shaheen | 2022 | not applicable | yes | yes | yes | no | yes | yes | yes | low risk |
| Kalinaki | 2022 | not applicable | yes | yes | yes | no | yes | yes | yes | low risk |
| Utomo | 2022 | no | yes | yes | no | no | unclear | yes | yes | high risk |
| GRIGOROVSKI | 2017 | not applicable | yes | yes | yes | unclear | yes | yes | yes | low risk |
| Rodrigo Blanco | 2013 | no | yes | yes | yes | unclear | yes | yes | yes | unclear |
| Yizhuo | 2014 | not applicable | yes | yes | no | no | unclear | yes | yes | unclear |
| Arazi | 2025 | not applicable | not applicable | not applicable | yes | no | yes | yes | yes | low risk |
| Kruger | 2022 | not applicable | not applicable | not applicable | yes | no | yes | no | yes | unclear |
| Shaheen | 2021 | not applicable | not applicable | not applicable | yes | no | yes | yes | yes | low risk |
| Jauquier | 2022 | not applicable | not applicable | not applicable | yes | no | yes | yes | yes | low risk |
| Wesevich | 2023 | not applicable | not applicable | not applicable | yes | no | yes | yes | yes | low risk |
| Alakaloko | 2022 | not applicable | not applicable | not applicable | yes | no | yes | yes | yes | low risk |
| Fufa | 2024 | not applicable | not applicable | not applicable | yes | no | yes | unclear | yes | unclear |
| Paintsil | 2015 | not applicable | not applicable | not applicable | yes | no | yes | unclear | unclear | high risk |
| Muulu | 2024 | not applicable | not applicable | not applicable | yes | unclear | yes | no | yes | unclear |
| Saghir Khan | 2024 | not applicable | not applicable | not applicable | yes | unclear | yes | yes | yes | low risk |
| Verma | 2016 | not applicable | not applicable | not applicable | yes | unclear | yes | yes | yes | unclear |
| Holmes | 2023 | not applicable | not applicable | not applicable | yes | yes | yes | yes | yes | low risk |
| Anyanwu | 2015 | not applicable | unclear | unclear | no | no | yes | unclear | no | high risk |
| Ekuk | 2023 | not applicable | not applicable | not applicable | yes | no | yes | unclear | yes | unclear |
| Rahiman | 2022 | not applicable | not applicable | not applicable | yes | unclear | yes | yes | yes | low risk |
| Bahoush | 2020 | not applicable | yes | yes | yes | yes | yes | yes | yes | low risk |
| Nasir | 2024 | not applicable | yes | yes | yes | unclear | yes | unclear | no | unclear |
| Uittenboogaard | 2021 | not applicable | yes | yes | yes | unclear | yes | unclear | yes | low risk |
| Hadley | 2013 | not applicable | yes | yes | yes | no | yes | unclear | yes | high risk |
| Ghafoor | 2020 | not applicable | yes | yes | yes | unclear | yes | yes | yes | low risk |
| Joseph | 2022 | not applicable | yes | yes | yes | no | yes | yes | yes | low risk |
| Adegoke | 2024 | not applicable | unclear | unclear | yes | no | no | unclear | no | high risk |
| Herrera-Toro | 2019 | not applicable | yes | yes | yes | yes | unclear | yes | yes | low risk |
| Isa | 2013 | not applicable | yes | yes | yes | yes | yes | yes | yes | low risk |
| Maher | 2014 | not applicable | no | no | yes | unclear | yes | yes | no | high risk |
| Qureshi | 2018 | not applicable | yes | yes | yes | unclear | yes | yes | yes | low risk |
| Seminara | 2019 | not applicable | yes | unclear | yes | unclear | yes | yes | unclear | unclear |
| Al-Jumaily | 2024 | not applicable | yes | yes | yes | yes | yes | yes | yes | low risk |
| Bukhari | 2023 | not applicable | yes | yes | yes | yes | yes | yes | yes | low risk |
| Zahir | 2023 | not applicable | yes | yes | yes | unclear | yes | yes | yes | low risk |
| Holmes | 2022 | not applicable | yes | yes | yes | yes | yes | yes | yes | low risk |
| Anwar | 2017 | not applicable | yes | no | unclear | no | yes | no | unclear | high risk |
| Geel | 2020 | yes | yes | yes | yes | yes | yes | yes | yes | low risk |
| Stanley | 2016 | not applicable | yes | yes | yes | yes | yes | no | yes | low risk |
| Kriel | 2019 | no | yes | yes | yes | unclear | yes | yes | yes | low risk |
| Stanley | 2018 | unclear | yes | yes | yes | unclear | yes | no | yes | unclear |
| Westmoreland | 2017 | not applicable | yes | yes | yes | unclear | yes | yes | yes | low risk |
| Luna-Fineman | 2023 | not applicable | yes | yes | yes | unclear | yes | yes | yes | low risk |
| Ramirez | 2024 | not applicable | yes | yes | yes | unclear | yes | yes | yes | low risk |
| Davidson | 2013 | not applicable | yes | yes | yes | yes | yes | yes | yes | low risk |
| DeBoer | 2020 | not applicable | yes | yes | yes | yes | yes | yes | yes | low risk |
| Geel | 2023 | not applicable | yes | yes | yes | yes | yes | yes | yes | low risk |
| Chauhan | 2021 | not applicable | yes | yes | yes | unclear | yes | yes | yes | low risk |
| Stanley | 2016 | not applicable | yes | yes | yes | yes | yes | no | yes | unclear |
| Madney | 2024 | not applicable | yes | yes | yes | yes | yes | yes | yes | low risk |
| Starling | 2024 | not applicable | yes | yes | yes | no | yes | yes | yes | low risk |
| Hazarika | 2023 | not applicable | yes | yes | yes | unclear | yes | yes | yes | low risk |
| Hamid | 2022 | yes | no | no | no | no | no | yes | yes | high risk |
| Swaminathan | 2008 | not applicable | no | no | yes | yes | yes | unclear | unclear | high risk |
| Pribnow | 2017 | not applicable | no | no | yes | no | yes | unclear | unclear | high risk |
| Ghafoor | 2019 | not applicable | yes | yes | yes | yes | yes | yes | yes | low risk |
| Karhan | 2019 | not applicable | yes | yes | yes | yes | yes | yes | yes | low risk |
| Marín | 2015 | not applicable | yes | yes | yes | unclear | yes | yes | yes | low risk |
| Silva | 2016 | not applicable | yes | yes | yes | no | yes | yes | no | high risk |
| Stulac | 2016 | not applicable | yes | yes | yes | no | yes | unclear | yes | unclear |
| Jabeen | 2016 | not applicable | yes | yes | yes | yes | yes | yes | yes | low risk |
| Suarez | 2015 | not applicable | yes | yes | yes | yes | yes | yes | yes | low risk |
| Jaime-P erez | 2017 | unclear | yes | yes | yes | unclear | yes | yes | yes | low risk |
| Pan | 2015 | not applicable | yes | yes | yes | unclear | yes | yes | yes | low risk |
| Geel | 2017 | not applicable | yes | yes | yes | yes | yes | yes | yes | low risk |
| Pe ́rez-Cuevas | 2012 | not applicable | yes | yes | yes | unclear | yes | yes | yes | low risk |
| Bravo | 2013 | not applicable | yes | yes | yes | not applicable | yes | yes | yes | low risk |
| Chapman | 2024 | not applicable | yes | yes | yes | no | yes | no | yes | high risk |
| Buckle | 2016 | not applicable | yes | yes | yes | unclear | yes | yes | yes | low risk |
| Mehreen | 2019 | not applicable | yes | yes | yes | unclear | yes | yes | yes | low risk |
| Sherief | 2015 | not applicable | yes | yes | yes | unclear | yes | yes | yes | low risk |
| Trehan | 2013 | not applicable | yes | yes | yes | unclear | yes | yes | yes | low risk |
| Srinivasan | 2020 | not applicable | yes | yes | yes | unclear | yes | yes | yes | low risk |
| Shamoon | 2018 | not applicable | yes | yes | yes | unclear | yes | yes | yes | low risk |
| Jain | 2016 | not applicable | yes | yes | yes | unclear | yes | yes | yes | low risk |
| San Roman | 2013 | not applicable | yes | yes | yes | no | no | no | yes | high risk |
| Denburg | 2019 | not applicable | yes | yes | yes | no | no | unclear | yes | high risk |
| Al-Jumaily | 2023 | not applicable | yes | yes | yes | unclear | yes | yes | yes | low risk |
| Belgaumi | 2016 | not applicable | yes | yes | yes | unclear | yes | yes | yes | low risk |
| Appeadu-Mensah | 2024 | not applicable | yes | yes | yes | unclear | yes | yes | yes | low risk |
| Uche | 2013 | no | yes | unclear | yes | no | unclear | yes | yes | high risk |
| Mutyaba | 2019 | not applicable | yes | unclear | yes | no | yes | no | yes | unclear |
| Arora | 2023 | not applicable | yes | yes | yes | no | unclear | yes | yes | high risk |
| Businge | 2024 | not applicable | no | no | yes | yes | yes | yes | yes | high risk |
| Parkin | 2020 | not applicable | no | no | yes | unclear | yes | yes | yes | high risk |
| Handayani | 2021 | not applicable | yes | yes | yes | yes | yes | yes | yes | low risk |
| Gibson | 2018 | not applicable | no | no | yes | unclear | yes | yes | yes | high risk |
| Pascual Morales | 2022 | not applicable | no | no | yes | unclear | yes | yes | yes | high risk |
| Stefan | 2014 | not applicable | yes | yes | yes | yes | yes | unclear | yes | unclear |
| Stones | 2014 | not applicable | unclear | unclear | yes | no | yes | no | yes | unclear |
| Farrag | 2023 | not applicable | yes | yes | yes | unclear | yes | unclear | yes | unclear |
| Elhassan | 2019 | not applicable | yes | unclear | yes | unclear | yes | unclear | yes | unclear |
| El-Mallawany | 2017 | not applicable | yes | yes | yes | yes | yes | no | yes | unclear |
| Sankara | 2020 | not applicable | yes | yes | yes | unclear | yes | unclear | yes | unclear |
| Slone | 2018 | not applicable | yes | yes | yes | unclear | yes | no | yes | unclear |
| Bordbar | 2023 | not applicable | yes | no | yes | yes | yes | yes | yes | low risk |
| Lopera Marín | 2015 | not applicable | yes | yes | yes | yes | yes | yes | yes | low risk |
| Machin Garcia | 2020 | not applicable | no | no | yes | no | yes | yes | yes | high risk |
| Guzmán | 2017 | not applicable | yes | yes | yes | no | yes | no | yes | high risk |
| Morosini | 2023 | not applicable | no | no | yes | no | yes | yes | yes | high risk |
| Lepe-Zúñiga | 2018 | not applicable | yes | yes | yes | no | yes | yes | yes | unclear |
| Sarmiento-Urbina | 2018 | not applicable | yes | yes | yes | yes | yes | yes | yes | low risk |
| Trujillo | 2016 | not applicable | yes | yes | yes | yes | yes | yes | yes | low risk |
| Villegas | 2013 | not applicable | yes | yes | yes | no | yes | yes | yes | low risk |
| Zouain-Figueiredo | 2013 | not applicable | unclear | no | yes | unclear | yes | yes | yes | unclear |
| Gaytan-Morales | 2018 | not applicable | yes | yes | yes | yes | yes | yes | yes | low risk |
| Chen | 2013 | not applicable | yes | yes | yes | unclear | yes | yes | yes | low risk |
| Trabelsi | 2014 | not applicable | unclear | yes | yes | no | yes | no | yes | high risk |
| Prasad | 2021 | no | unclear | yes | yes | no | yes | yes | yes | unclear |
| Stagno | 2013 | no | no | yes | yes | no | unclear | yes | yes | unclear |
| Ribeiro Lima | 2015 | not applicable | yes | yes | yes | no | yes | yes | yes | low risk |
| Uche | 2021 | not applicable | yes | no | yes | no | unclear | yes | yes | high risk |
| Moleti | 2019 | yes | yes | yes | yes | unclear | yes | yes | yes | low risk |
| Martijn | 2017 | not applicable | yes | no | unclear | no | unclear | yes | yes | high risk |
| Egypt | **2021** | not applicable | yes | yes | yes | no | yes | unclear | yes | unclear |

JBI criteria in case-control studies

| **Author** | **Year** | **Were cases and controls comparable?** | **Were cases clearly defined?** | **Were controls clearly defined?** | **Was exposure measured reliably?** | **Were confounding factors identified and managed?** | **Were risk factors identified and measured appropriately?** | **Were statistical analyses appropriate?** | **Overall appraisal** |
| --- | --- | --- | --- | --- | --- | --- | --- | --- | --- |
| Sevilla-Castillo | 2017 | unclear | yes | no | yes | unclear | unclear | no | high risk |
| Viana | 2015 | unclear | yes | yes | yes | unclear | unclear | unclear | unclear |
| Aronson | 2014 | unclear | yes | yes | yes | unclear | unclear | unclear | unclear |

JBI criteria in cross-sectional studies

| **Author** | **Year** | **Were inclusion criteria clearly defined?** | **Was the population representative?** | **Were exposures identified and measured appropriately?** | **Were outcomes identified and measured appropriately?** | **Were statistical analyses appropriate?** | **Overall appraisal** |
| --- | --- | --- | --- | --- | --- | --- | --- |
| de Souza Silva | 2021 | yes | yes | NA | yes | yes | low risk |
| Grace | 2024 | no | no | yes | yes | yes | unclear |
| Dufort | 2021 | yes | yes | yes | yes | yes | low risk |
| Sherief | 2015 | yes | yes | yes | yes | yes | low risk |
| Varghese | 2018 | yes | yes | yes | yes | yes | low risk |
| Smith | 2024 | yes | yes | yes | yes | yes | low risk |
| Brito Silveira | 2023 | yes | yes | yes | yes | yes | unclear |
| Herrera-Toro | 2020 | unclear | yes | yes | yes | yes | low risk |

JBI criteria in clinical trials

| **Author** | **Year** | **Was randomization used (if applicable)?** | **Was allocation concealment used?** | **Were the groups comparable at baseline?** | **Was blinding used for participants and/or researchers?** | **Were outcome measures reliable and standardized?** | **Was the follow-up period adequate?** | **Were confounding factors identified and managed?** | **Were statistical analyses appropriate?** | **Overall appraisal** |
| --- | --- | --- | --- | --- | --- | --- | --- | --- | --- | --- |
| Pongtanakul | 2020 | non applicable | non applicable | yes | non applicable | yes | yes | unclear | yes | high risk |
| Stary | 2014 | Yes | Unclear | Yes | Unclear | Yes | yes | yes | yes | low risk |
| Silvia Regina Brandalise | 2016 | yes | unclear | yes | no | yes | yes | yes | yes | low risk |
| Elzomor | 2017 | NA | NA | NA | NA | yes | yes | yes | yes | high risk |
| Rojanaporn | 2019 | NA | NA | NA | NA | yes | yes | yes | yes | high risk |
| Yao | 2019 | no | unclear | unclear | unclear | yes | yes | no | yes | unclear |
| Bouda | 2019 | unclear | unclear | yes | unclear | yes | no | yes | yes | unclear |
| Brandalise | 2016 | Yes | unclear | yes | unclear | yes | yes | yes | yes | low risk |
| Chagaluka | 2020 | NA | NA | NA | NA | yes | yes | unclear | yes | high risk |
| Radhakrishnan | 2023 | NA | NA | NA | NA | yes | yes | unclear | yes | high risk |
